# Yellowstone Hot Springs are Organic Chemodiversity Hot Spots

**DOI:** 10.1038/s41598-018-32593-x

**Published:** 2018-09-20

**Authors:** Michael Gonsior, Norbert Hertkorn, Nancy Hinman, Sabine E.-M. Dvorski, Mourad Harir, William J. Cooper, Philippe Schmitt-Kopplin

**Affiliations:** 1 University of Maryland Center for Environmental Science, Chesapeake Biological Laboratory, Solomons, Maryland USA; 2 Helmholtz Zentrum Muenchen, German Research Center for Environmental Health, Neuherberg, Germany; 3 University of Montana, Department of Geosciences, Missoula, USA; 4 University of California Irvine, Department of Civil and Environmental Engineering, Irvine, USA; 5 Technische Universität München, Institute for Analytical Food Chemistry, Freising-Weihenstephan, Germany

## Abstract

Yellowstone National Park hydrothermal springs were investigated according to their organic geochemistry with a special focus on the Yellowstone hot spring dissolved organic matter (YDOM) that was solid-phase extracted. Here we show that YDOM has a unique chemodiversity that has not yet been observed anywhere else in aquatic surface environments and that Yellowstone hot springs are organic chemodiversity hot spots. Four main geochemically classified hot spring types (alkaline-chloride, mixed alkaline-chloride, acid-chloride-sulfate and travertine-precipitating) exhibited distinct organic molecular signatures that correlated remarkably well with the known inorganic geochemistry and manifested themselves in excitation emission matrix fluorescence, nuclear magnetic resonance, and ultrahigh resolution mass spectra. YDOM contained thousands of molecular formulas unique to Yellowstone of which 80% contained sulfur, even in low hydrogen sulfide containing alkaline-chloride springs. This unique YDOM reflects the extreme organic geochemistry present in the hydrothermal features of Yellowstone National Park.

## Introduction

Physical, chemical, and biological processes control the evolution of minerals and dissolved organic matter (DOM) over a vast range of mass, length, and time^1^. Indeed, mutual interferences and interactions among these processes presumably have defined chemical and biological evolution throughout Earth history. Modern terrestrial biology operates largely within limited temperature, pH, and redox conditions. In contrast, extreme environments, such as hot springs, have wide ranges of physicochemical parameters, generally believed to be similar to environments found on early Earth^2^. Since then, chemical, mineral, and especially biological processes have shaped organic matter diversity on Earth^1^. Yet, little is known about the organic composition, specifically the chemical diversity of DOM in geothermal systems, despite some characterization of marine hydrothermal waters^3,4^. In the case of some Yellowstone hot springs, DOM, such as petroleum products^5^, or microbial metabolites such as lipids^6^ have been previously identified. The incorporation of sulfur into DOM also needs to be considered because of potential reactions between DOM and hydrogen sulfide that is often present in hydrothermal features, and because hydrosulfurization of DOM has been previously suggested in hydrothermal vents^3^. However, dissolved organic sulfur (DOS) in Yellowstone hot springs has not been characterized to date. The dissolved organic nitrogen (DON) also remains largely uncharacterized at the structural level in aquatic systems, despite the characterization of small biomolecules such as urea^7^ and dissolved combined amino acids (DCAA)^8^, which only account for about 10% of the DON pool. The often used solid-phase extraction of DOM also appears to have a limited extraction efficiency for DON, which makes it difficult to comprehensively evaluate DON. However, DON extraction efficiencies are not known for DON in hydrothermal systems. To the best of our knowledge, no data exists on any DON components in hydrothermal systems.

The Yellowstone Plateau, the youngest in a line of volcanic calderas stretching along the length of the Snake River Plain and dating back 17 million years^9^, exhibits abundant and diverse hydrothermal features that are ideal for studying DOM in extreme environments. The caldera hosts an extensive hydrothermal system, which cools the shallow underlying magma body^10,11^. The precise details of the magmatic system are still debated, but recent reports suggest that an underlying basaltic magma heats an overlying rhyolitic magma^11^. The shallower, rhyolitic magma provides the heat that drives hydrothermal circulation. The contributions of this thermal exchange to the inventory of inorganic salts in the hydrothermal system have recently been better established. A caldera-wide analysis of river composition and discharge^12^ suggested that ~40% of the dissolved solids are derived from acid hydrolysis of country rock by acidic magmatic gases. Still, variable concentrations of gases and solids are observed in the numerous thermal features (hot springs, geysers, mud pots, fumaroles, and acid lakes) that are spread throughout the caldera (e.g. Firehole drainage thermal areas) and just outside its margins (e.g. Norris Geyser Basin), suggesting differences in sources of materials and in processes of fluid and gas evolution. The intra-caldera hydrothermal system may serve to enhance adjacent thermal systems at Mammoth Hot Springs, north of the main hydrothermal system^13^. Extra-caldera hydrothermal systems would be expected to have different sources of dissolved constituents.

In 1966, Thomas Brock discovered microorganisms in the boiling hot springs, and their outflow channels, of Yellowstone National Park^14^. This remarkable observation raised many new questions. Among these was the question of what carbon and energy sources are available to support heterotrophic extremophiles. Furthermore, *in situ* metabolic processes of the autotrophic and heterotrophic microbial communities in hot springs are largely not understood, despite advances in our understanding of the microbial community composition^15–17^ as well as the inorganic geochemical and mineralogical composition of the thermal waters^10^. This is further compounded by our fundamental lack of understanding of the diversity and abundance of organic compounds in these springs.

Hawke *et al*.^18^ showed that DOM is largely lost during geothermal heating but did not address changes in organic chemical composition in the remaining material. In Yellowstone hot springs, DOM is leached from deeply buried sedimentary rock^5^, further confounding the sources and composition of DOM. Different subsurface flow paths for heated water and gases define the inorganic geochemical properties of individual springs^10^, likely influencing DOM composition as well. Herein, we address the question of chemodiversity of hot spring DOM and approach the possible processes responsible for the specific composition of Yellowstone hot spring DOM (YDOM). A classification of types of hot springs based on the YDOM composition is also evaluated.

## Results

### Inorganic and organic geochemical comparison of hot spring types

**Figure 1:**
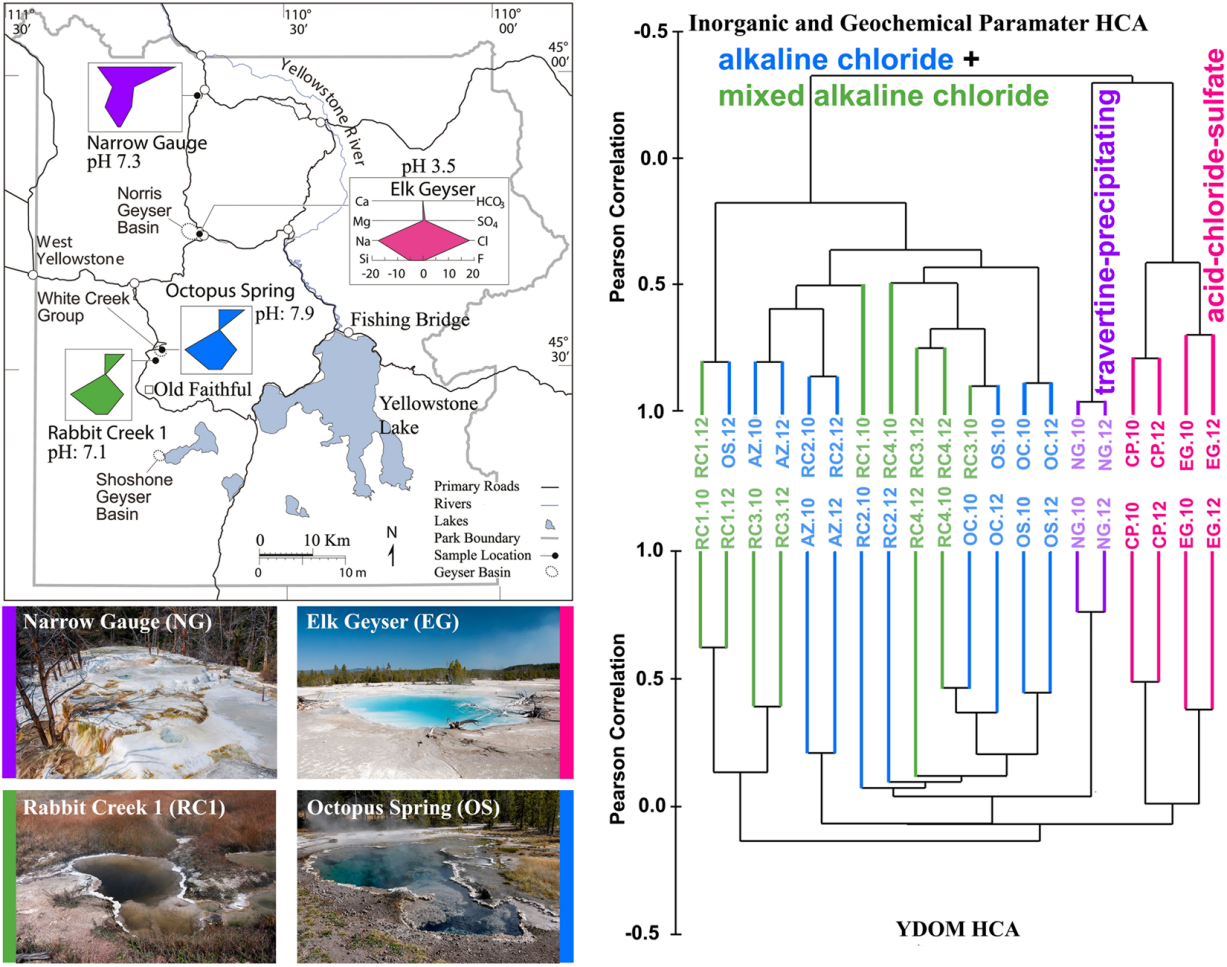
Map of Yellowstone National Park including stiff diagrams of key inorganic ion abundances in hot springs (top, left); photographs of Narrow Gauge Spring (**NG**), Rabbit Creek (**RC1**), Elk Geyser (**EG**) and Octopus Spring (**OS**) (bottom, left); hierarchical cluster analyses (HCA) of inorganic and geochemical parameters (top, right) (see also Supplementary Table. S1); and YDOM (bottom, right). Note: additional Yellowstone hot springs were used for hierarchical cluster analyses: Azure (**AZ**), Ojo Caliente (**OC**), Rabbit Creek 3 and 4 (**RC3** and **RC4**), and Cinder Pool (**CP**). Details about all springs can be found in the supplementary online material. Replicate samples were collected in 2010 and 2012 (e.g. NG.10 and NG.12). All photographs were taken under Yellowstone Research Permit YELL-2017-SCI-5828.

The selected 10 Yellowstone hot springs were analyzed with respect to their inorganic geochemistry and physical properties and clustered into four compositional groups: travertine-precipitating, mixed-alkaline-chloride, alkaline-chloride, and acid-chloride-sulfate springs (Fig. 1 and Supplementary Table. S1). Out of these, four representative hot springs were investigated in detail with respect to DOM composition, namely Narrow Gauge (**NG**), Mammoth Hot Spring Complex (travertine-precipitating); Rabbit Creek (**RC1**), Midway Geyser Basin (mixed (terrestrial) alkaline-chloride); Elk Geyser (**EG**), Norris Geyser Basin (acid-chloride-sulfate); and Octopus Spring (**OS**), Lower Geyser Basin (alkaline-chloride) (Fig. 1). To describe in detail this DOM, we used electrospray ionization Fourier transform ion cyclotron resonance (FT-ICR) mass spectrometry and high field nuclear magnetic resonance (NMR) spectroscopy. It should be noted here that solid-phase extracted material was used for FT-ICR MS and NMR analyses and hence very hydrophilic compounds are not extracted and lost. Furthermore, the analytical window of FT-ICR MS is biased towards the strongest ionizing components in a complex mixture and hence is likely not representative of the whole DOM pool. Nevertheless, these techniques are information-rich methods to accurately depict the molecular diversity of polydisperse and molecularly heterogeneous DOM by identifying the ionizable composition (FT-ICR) and structure (NMR) of solid-phase extractable DOM^19,20^. Additionally, Excitation Emission Matrix (EEM) fluorescence was used to describe the fluorophores present in these springs.

### YDOM composition in hot spring types

**Figure 2:**
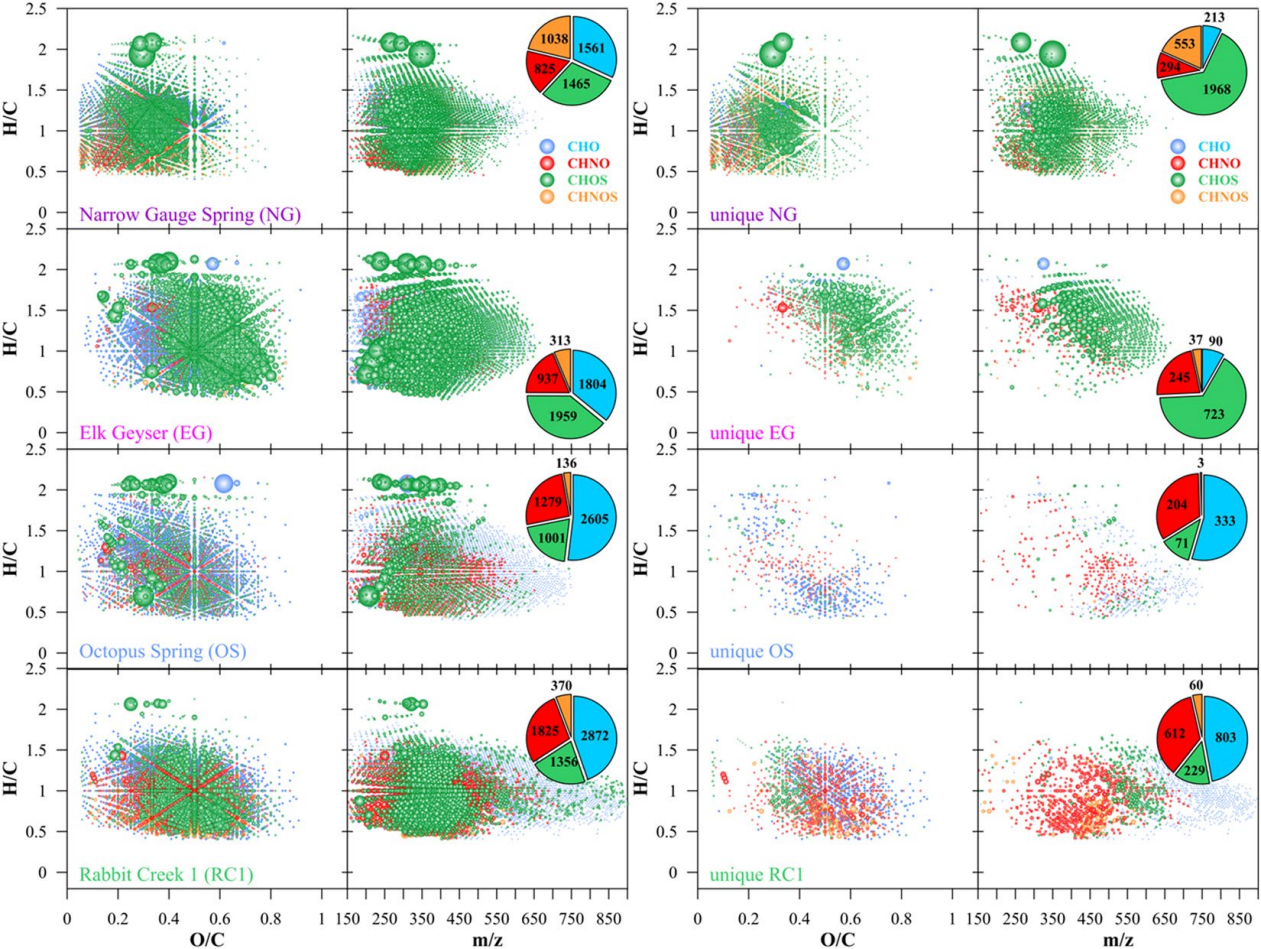
Detailed FT-ICR MS results (van Krevelen diagrams and mass-edited H/C ratio plots) for representative springs of each of the four compositional groups (see also clusters in Fig. 1): Narrow Gauge Spring (NG, travertine-producing), Octopus Spring (OS, alkaline chloride), Elk Geyser (EG, acid-chloride-sulfate) and Rabbit Creek (RC1, mixed alkaline chloride) and their unique molecular signatures, respectively. Note: Bubble area reflects relative abundance of *m/z* ions; color code of CHO, CHNO, CHOS and CHNOS molecular series according to figures.

These complementary techniques demonstrated unprecedented organic molecular diversity of DOM in individual Yellowstone hot springs (Fig. 2 and Supplementary Figs S1–S8). FT-ICR MS, with its excellent mass resolution and mass accuracy, allows unambiguous assignment of thousands of molecular formulas directly out of complex mixtures, including determination of molecular formulas containing heteroatoms like nitrogen and sulfur^21^. Results from FT-ICR MS showed that organic molecular compositions statistically clustered with the grouping of the 10 springs by inorganic chemistry (Fig. 1) yet also demonstrated distinct DOM compositions in the four individual springs studied in more detail (Fig. 2).

**Figure 3:**
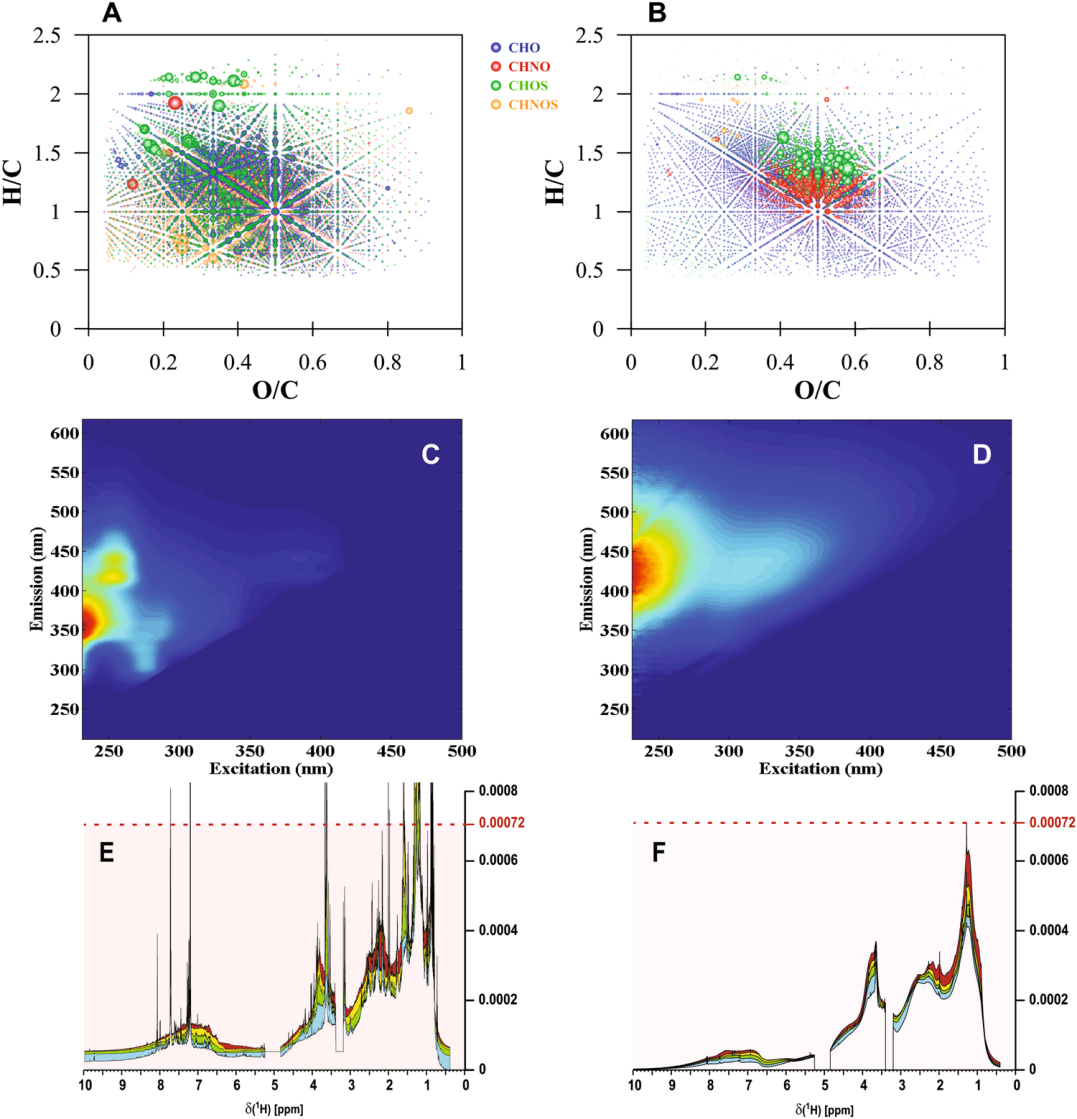
Van Krevelen diagrams of four (NG, EG, RC1, OS) consolidated YDOM samples (**A**) compared to consolidated solid phase extracted (SPE)-DOM samples from 114 different aquatic systems (**B**) (see details in supplementary online material). Consolidated YDOM EEM spectrum (**C**) and ^1^H-NMR spectrum in comparison to a consolidated EEM spectrum in ultrapure water and ^1^H-NMR data (**F**) of four Swedish boreal lake SPE-DOM samples. Note: Bubble area in van Krevelen diagrams reflects relative abundance of *m/z* ions.

NMR offers quantitative and non-destructive determination of chemical environments for carbon and hydrogen and is particularly useful in depicting aliphatic groups based on sp^3^-hybridized carbon. NMR results documented that the structure of YDOM is substantially different compared to that of boreal lakes (Fig. 3) and other aquatic systems^19,22,23^.

EEM fluorescence spectroscopy has been frequently used to describe the chromophoric dissolved organic matter in aquatic systems^24–27^. Remarkably, each spring type contained different fluorophores that were also not found in other surface waters (Supplementary Fig. S8). Fluorophores are in general indicative of conjugated aromatic π-electron systems that absorb in the ultraviolet and emit light in the visible spectrum. To further describe the unique nature of YDOM, results from each analytical technique are presented in greater detail below.

### YDOM characterization using non-target FTICR Mass Spectrometry

Ultrahigh-resolution FT-ICR mass spectra of the four YDOM from the representative springs (**NG**, **EG**, **RC1**, and **OS**) provided several thousand *m/z* peaks (Supplementary Fig. S1) of which many were assigned to extended molecular formula series containing atomic combinations of carbon, hydrogen, and oxygen (CHO), containing nitrogen (CHNO), containing sulfur (CHOS), and containing both sulfur and nitrogen (CHNOS) (Fig. 2, Supplementary Table. S2). The precisely determined exact molecular formulas (to < 0.0001 amu from theoretical formula) represented a remarkably wide coverage of the compositional space that is shown here in van Krevelen diagrams or elemental plots, where the oxygen to carbon ratio (O/C) is plotted against the hydrogen to carbon ratio (H/C) of each individual molecular formula (Supplementary Figs S2, S3). We define chemodiversity by using the coverage of assigned molecular formulas in van Krevelen space, the molecular weight distribution and numbers of assigned formulas and hence van Krevelen and mass–edited H/C plots can be used to visualize the very high chemodiversity of the YDOM.

In essence, mixed-alkaline-chloride and alkaline-chloride springs (**RC1**, **OS**) had diverse unsaturated or aromatic (H/C < 1.5) molecular ions, but also saturated CHOS molecular formulas up to 800 *m/z* (Fig. 2 and Supplementary Figs S2–S4), whereas travertine-precipitating hot springs (**NG**) were enriched in hydrogen-deficient nitrogen and sulfur-containing low molecular weight molecules (H/C < 1.5; *m/z* < 500) with limited oxygenation (O/C < 0.5). Acid-chloride-sulfate springs (**EG**) had the widest diversity of unique molecular ions from small to intermediate *m/z* (150 < *m/z* < 650; Fig. 2 and Supplementary Figs S2–S4). The hot springs **NG** and **EG** had the most sulfur-containing molecular formulas with a remarkable coverage of the chemical space shown within the van Krevelen diagrams (Fig. 2 and Supplementary Figs S2–S4). Spring **RC1** had the highest number of nitrogen-containing molecular ions, which were mostly unsaturated and/or aromatic in nature (H/C < 1.5).

Hierarchical clustering of the FT-ICR MS-derived YDOM chemical compositions (Fig. 1, bottom) showed statistically significant correlation with the previously well-documented inorganic chemistry of geothermal-water types (e.g.^10^; Fig. 1 and Supplementary Table. S1). The inorganic constituents derive from the interaction of hydrothermal water with country rock, coupled with retention or release of the water-soluble gases, CO_2_ and H_2_S, the latter of which oxidizes to form H_2_SO_4_. The observed congruence of inorganic and organic compositions suggested a decisive influence of physico-chemical and mineral conditions on the synthesis of YDOM, which may be causal in that the inorganic constituents are intimately involved in transformations of YDOM and which might be further mediated by microbiological processes^17^. Consequently, the four representative springs contain distinctly different YDOM (Supplementary Figs S2 and S3).

**Figure 4:**
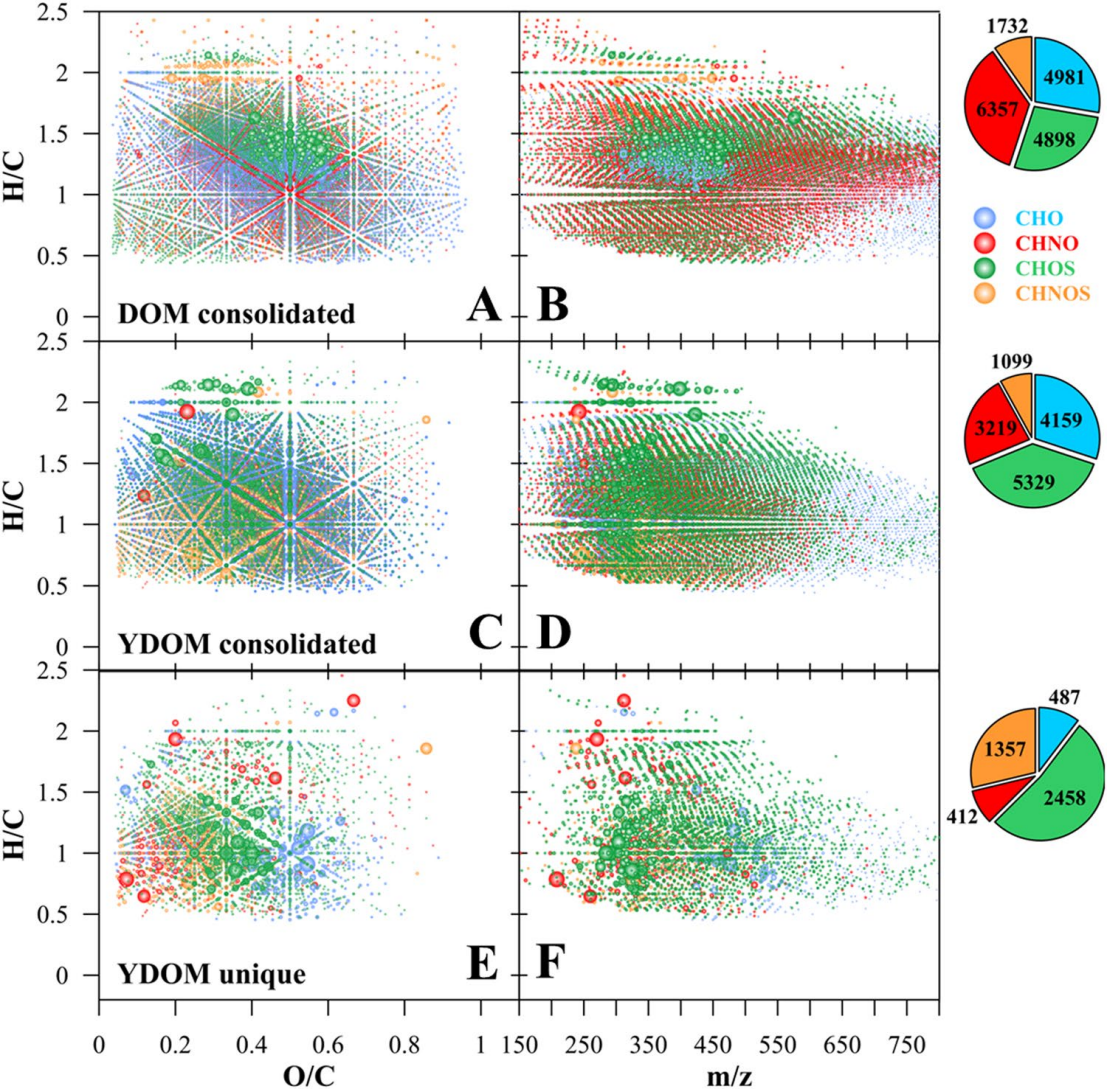
Comparison of consolidated SPE-DOM molecular signatures (**A**,**B**) analyzed by non-targeted FT-ICR MS and collected on a large spatial scale globally and consolidated YDOM signatures (**C**,**D**), including the unique signatures isolated from this comparison (**E**,**F**). Left column (**A**,**C**,**E**): van Krevelen diagrams; right column (**B**,**D**,**F**): mass-edited H/C ratio plot; pie diagrams: counts of assigned elemental compositions. Note: Bubble area reflects relative abundance of *m/z* ions.

To compare YDOM to the composition of other types of DOM from conventional aquatic systems, we contrasted FT-ICR mass spectra of our representative set of four hot spring YDOM samples with an extensive compendium of lake, river, estuarine, and marine DOM (n = 114) (Figs. 3 and 4) that had been isolated by the same solid-phase extraction procedure (SPE-DOM) and analyzed to the same *m/z* resolution. We found that the consolidated YDOM data set and that of aquatic surface waters, which both showed >10^4^ assigned molecular formulas, were largely dissimilar. For example, YDOM from the four geochemical systems showed 5,405 unique molecular formulas that were not present in the consolidated data set for other aquatic SPE-DOM (Figs 3, 4 and Supplementary Table S3). Furthermore, the majority of the unique YDOM molecular formulas contained heteroatoms such as nitrogen and sulfur (~9% CHNO; ~52% CHOS; ~28% CHNOS compounds; Fig. 4).

### YDOM characterization using Nuclear Magnetic Resonance (NMR) Spectroscopy

Chemodiversity of YDOM can also be expressed using quantitative and structural NMR spectroscopy. For comparison with YDOM, we used boreal lake SPE-DOM (collected in central Sweden, Malingsbo region) because boreal lake DOM is largely representative of diverse aquatic surface DOM^19,22,28,29^. Boreal lake SPE-DOM had a continuous distribution of broad ^1^H NMR resonances (Fig. 3) that reflected massive superposition of >10^6^ individual atomic environments found in common DOM^19,22,30^. In contrast, YDOM showed better-resolved groups of NMR resonances across the entire chemical shift range and considerable distinction among the four selected hot springs (Supplementary Fig. S4). Hence, ^1^H NMR spectra supported the chemical diversity observed in FT-ICR MS and confirmed a remarkable structural diversity within YDOM throughout all classes of chemical bonds (Supplementary Figs S4–S7, Tables S5–S7; see detailed NMR discussion in Supplementary text) along with fundamentally different structural distributions when compared to common aqueous and boreal lake SPE-DOM.

Aliphatic and aromatic structures were observed to varying degrees in YDOM. The four hot springs had significant amounts of aliphatic structures, although the configuration varied between them. YDOM from **RC1** and **NG** was enriched in C_3–5_ branched, open-chain aliphatic compounds, terminated by methyl and carboxylic groups, whereas alicyclic rings such as refractory carboxyl-rich alicyclic molecules (CRAM)^28^ dominated **OS** and especially **EG** (Supplementary Fig. S4), suggesting different formation mechanisms and/or precursor composition. Aromatic functional groups (C_ar_H) with a chemical shift in δ_H_ of 9–6.5 ppm in YDOM fell into two groups representing aromatic ethers and ketones (Supplementary Figs S5b, S6b). C_ar_H shifts were also different from boreal SPE-DOM, reinforcing the unique character of YDOM. **EG** had a near Gaussian distribution of aromatic NMR resonances, indicating an even abundance of electron-withdrawing, neutral, and electron-donating functional groups (Supplementary Figs S5, S5b, S6b). A ramp-like increase of aromatic hydrogen from lower to higher chemical shift δ_H_ indicated abundant polyphenolic compounds and/or aromatic ethers decreasing in the order **OS >> RC1 > NG**, suggesting variable contributions from terrestrial organic matter and (poly)phenols (Supplementary Figs S4, S5b, S6b).

In general, aliphatic functional group abundance and diversity (δ_H_ < ~3 ppm) decreased with decreasing pH (Supplementary Figs S5a, S6a, S7). Despite expected hydrolytic degradation of methoxy groups and oxomethylene structures in aliphatic compounds at elevated temperatures, such groups were markedly diverse in YDOM (Supplementary Fig. S8). Overall, the proportion of open-chain, branched aliphatic compounds decreased in the order **RC1** > **NG** > **OS** > **EG**.

Expected small contributions of heteroatomic functional groups are more difficult to determine in complex ^13^C and ^1^H NMR spectra, however **EG** also showed a unique set of presumably thiomethyl groups that is highlighted in circle b in Supplementary Fig. S6a.

Overall, NMR results are in very good agreement with results presented by using FT-ICR MS and support the conclusion that YDOM is unique when compared to other surface aquatic systems and that each individual spring type contains distinctly different YDOM.

### YDOM characterization using Excitation Emission Matrix (EEM) Fluorescence

The optically active fraction of YDOM also showed unique chemical signatures compared to boreal lake SPE-DOM (Fig. 3 and Supplementary Fig. S8) and other very diverse aquatic systems^26,31–33^. Maxima of the consolidated YDOM EEM fluorescence peaks of the combined four springs were located at very different excitation/emission couples (Fig. 3). The highest intensity local maximum of the consolidated YDOM dataset was located at an excitation|emission (Ex|Em) couple 230 nm|355 nm, which has not been observed in any aquatic surface SPE-DOM samples. However, to describe different fluorophores, individual EEM spectra need to be used and several distinct fluorescence peaks were observed in individual YDOM springs (Supplementary Fig. S8) (NG: Ex|Em = 240|360 nm, 260|420 nm; EG: Ex|Em = 230|310 nm, 230|410 nm; RC1: Ex|Em = 230|310 nm, OS: Ex|Em = 230|310 nm), representing rather distinct fluorophores of yet unknown provenance, except for peak 260|420 nm in NG, which is similar to the previous defined A peak or humic-like fluorescence^34^. It appears that there might be similar fluorophores present in EG, RC1 and OS with additional fluorophores at higher emission wavelengths in EG. NG appeared to be very different from all other springs, which may reflect the different source of the hydrothermal water. In agreement with NMR and FT-ICR mass spectra, the EEM fluorescence spectra of the four individual hot springs also showed clear distinction between these representative hot springs (Supplementary Fig. S8) and clear differences to SPE-DOM collected from diverse surface waters.

### Unique Chemodiversity of YDOM

**Figure 5:**
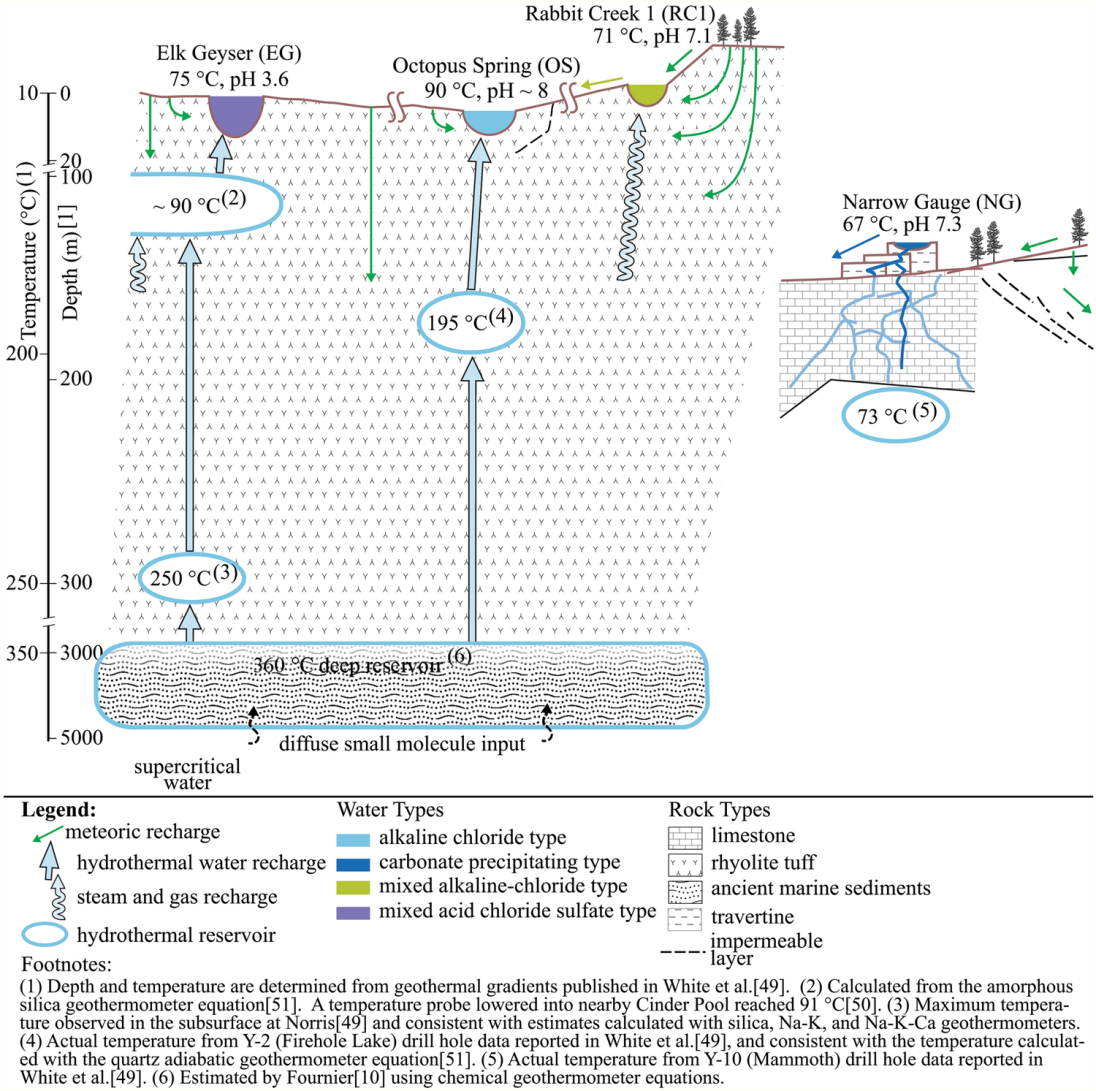
Conceptual diagram of YDOM transformation in Yellowstone hot springs as derived from drill core studies.

Combined results from these complementary analytical techniques demonstrated that the extensive compositional diversity as well as the structural specificity of YDOM make Yellowstone National Park hot springs DOM chemodiversity hotspots and further, that their DOM is largely indigenous to the geochemical types of hot springs. DOM arising from thermophiles will contribute to the observed chemodiversity. However, the altogether limited microbial abundance, and the all in all restricted microbial biodiversity observed in these extreme environments (Supplementary Table S4) likely cannot account for the observed molecular complexity of YDOM. This is also supported by the EEM fluorescence data, because only very weak signals that indicated the presence of proteins were found, which typically show a fluorescence signal at higher excitation wavelengths of either Ex|Em = 275|300 nm (tyrosine) or Ex|Em = 280|345 nm (tryptophan)^35^. Therefore, alternative sources of DOM must contribute to the diversity of organic molecules. While atmospheric deposition into small surface area springs in the pristine environments of Yellowstone National Park will likely be negligible, contributions from small molecules from deep thermal waters (>350 °C) are clearly conceivable. At these elevated temperatures and pressures, complex DOM is likely to decompose into small, and often oxygenated, molecules^18^. A proportion of this thermally transformed DOM is expected to be non-extractable^18^ and hence would escape our analysis in this study. Surface water runoff and groundwater are possible additional sources of DOM to these springs (Fig. 5).

While surface water DOM would resemble common SPE-DOM of rivers and lakes, groundwater may become severely transformed before entering the spring aquifers, depending on underground exposure to temperature, pressure, gases, and minerals. In the subsurface, gases (e.g. HCl, H_2_S, SO_2_, NH_3_) and minerals (e.g. clays, minerals^36^, and redox-active transition-metal compounds^37^) will become quite reactive reaction partners and catalysts^38^ for transformation of aqueous DOM at shallow depths, which is feasible for **EG**, considering that groundwater does not enter in Norris Geyser Basin deeper than 100 m^39^. Commonplace surface runoff DOM entering the springs will be processed at these near-boiling conditions, but it is not clear whether or not this terrestrially derived DOM can be so severely transformed in individual hot springs as to create the observed unique YDOM, especially when residence times of water in hot springs are low which is certainly the case for **NG** with no exposed pool and **OS** with a rather large discharge of an estimated 30 (base discharge) to 192 (surge discharge) L s^−1^ ^40^.

The molecular complexity of YDOM is likely to be influenced by several factors, once superheated fluids reach the surface. These factors may include both abiotic (e.g. redox- and photochemistry) and biotic (e.g. transformation of plant-derived DOM) processes that jointly define the environments. To further assess likely contributing sources of DOM, we evaluated each spring for possible contributions from marshes (runoff) and groundwater and put these contributions into context with direct hydrothermal sources to each spring (alkaline-chloride, acid-sulfate, and steam-condensates) (Table 1).

**Table 1:** Major and minor sources that contribute to the organic complexity in different types of hot springs in Yellowstone (major source (y**); minor source (y*); no contribution (n)).

spring name	surface runoff	ground-water	alkaline-chloride	acid-sulfate	steam-heated
RC1^a^	y**	n	y*	n	y*
EG^b^	n	y*	y**	y**	n
OS^c^	y*	n	y**	n	n
NG^d^	n	y**	n	n	n

Note: ^a^Based on visual observations of surface water input; [Si], [Cl^−^], and [Na^+^], which are traditional alkaline-chloride water components^10^; and slightly lower pH in the absence of significant [SO_4_^2−^], which is an indication of steam input^10^. ^b^Based on [Si], [Cl^−^], and [Na^+^], which are traditional alkaline-chloride water components^10^, low pH and high [SO_4_^2−^], and reported contributions of ~10% “cold, shallow, young” water^39^. ^c^Based on topographic observations (OS is at the base of a hill); [Si], [Cl^−^], and [Na^+^], which are traditional alkaline-chloride water components^10^; and reports of possible groundwater exchange^41^. ^d^Based on topographic observations (NG is at relatively high elevation above forest floor) and previous hydrological investigations^66^.

White Creek (locus of OS) and Norris Geyser Basin (locus of EG) are among the few areas for which groundwater – surface water interactions have been studied (White Creek^41^; Norris Geyser Basin^39,42^). Gibson and Hinman^41^ concluded that there is some potential for episodic exchange across a leaky sinter barrier between the adjacent marsh and Octopus Spring but did not provide an estimate. Gardner *et al*.^39^ concluded, based on tritium and CFC analyses, that 10% of “cold, shallow, young” water mixes with hydrothermal water at Norris Geyser Basin. It is worth noting that they comment on the complexity of the groundwater system at Norris Geyser Basin and therefore, the amount of potential mixing at EG might vary significantly from this value.

A conceptual diagram of the Yellowstone hydrothermal system illustrates how the surface and subsurface water flow paths connect water and vapor with the four springs in this study (Fig. 5) and highlights the potential for transformation of organic matter at different depths and corresponding temperatures^43^. The general circulation model for the Yellowstone caldera posits recharge from meteoric water at high elevations or through fractures within and across the rim of the caldera, as well as recirculation within the local hydrothermal systems^10,12,42,44–47^. Some of the DOM therefore likely originates through downward leaching of the regionally thin soils^48^ and organic-poor volcanic rocks. Most of the DOM in hot springs directly connected to deep sources (intracaldera, **OS** and Mammoth Hot Springs, **NG**) must be generated through internal reactions of DOM of these three sources: deep hydrothermal water, intra-caldera recirculated water, and infiltrating meteoric water, whereas DOM in the other springs (**RC1** and **EG**) is acquired at shallower depths and lower temperatures. Each spring, therefore, bears an organic geochemical signature dictated in part by the source of the water and partly from reactions that occur in all hydrothermal systems.

**OS** receives almost all of its water and solutes directly from a deep hydrothermal source (~195 °C and ~150 m depth^49^ (Fig. 5)), most closely representing an endmember among our sites for both organic and inorganic components in the hydrothermal system. In addition to deep hydrothermal water, **EG** receives groundwater from DOM-containing surface recharge, acidic gases, and steam from the underlying deep aquifer (below 23 m depth^49,50^). This acidified shallow groundwater also leaches solutes from bedrock. In contrast, **RC1** is disconnected from the deep aquifer that feeds **OS**. Instead, water and heat come from steam condensate that substantially interacts with bedrock and perhaps shallow groundwater, as well as DOM-containing water from surface runoff. **NG** is directly connected to a separate limestone aquifer source, which is absent from the caldera, with a theoretical temperature of ~110 °C based on a quartz adiabatic geothermometer calculation^51^. However, the actual temperature from the nearby Mammoth drill core Y10 maintained a constant temperature of 70 °C from ~15 m below the surface to the maximum depth of the drill hole at ~135 m^49^.

Temperature estimates from drill hole geothermal gradients^49^ revealed variations of depth at which a temperature of 350 °C, the proposed temperature of the parent thermal water^10^ and near supercritical conditions (374 °C), would be reached, ranging from 690 m (**NG**; drill hole Y10) > 570 m (**EG**; drill hole Y12) ≈ 560 m (**RC1**; drill hole Y5) > 470 m (**OS**; drill hole Y2). The depth at which supercritical conditions for aqueous fluids would be reached is too shallow to allow contributions of organic molecules from the marine rocks deeply buried (~3 km) under the caldera except for very small molecules capable of surviving these extreme conditions^18^. Circulating upwards, this deep hydrothermal water then accumulates in shallower aquifers (~150 m depth) where subsequent modifications of DOM may occur. The reactions that occur in the shallower aquifers are the ones that substantially modify the composition of hot spring water in individual springs.

Additional reactions, though, contribute to the diversity of organic molecules, where temperature, dissolved gases, and water-property changes play a critical role^52,53^. For example, the high number of unique sulfur-containing molecular ions in YDOM and specifically in the **NG** and **EG** samples is likely a result of reactions of DOM with hydrogen sulfide^54^. However, a distinct precursor pool of organic molecules must exist to create these unique molecular signatures. Indeed, for each YDOM sulfur-containing formula (CHOS), there is a feasible precursor CHO formula. These molecular signatures are also very different from DOM hydro-sulfurized under anaerobic conditions in sediments or soils (Supplementary Fig. S9). A comparison of YDOM sulfur-containing formulas with those of a sulfate-rich anaerobic-sediment pore-water DOM collected in the Chesapeake Bay (38°20′54.19″N, 76°18′47.34″W) revealed only a small overlap [94 (~4%) out of 2,458 CHOS formulas] despite a very high diversity of CHOS molecules present in the sediment pore water (1,703 CHOS formulas), further highlighting the unusual and largely unique nature of YDOM (Supplementary Fig. S9).

Geochemically formed organic molecules likely are produced from supercritical CO_2_ in deep-reaching tectonic faults^55,56^ and in alkaline hydrothermal vents^57^. Laboratory-based experiments confirmed extensive transformation reactions of hydrocarbons below 300 °C and 100 MPa pressure at hydrothermal conditions and document that complex organic molecules were indeed produced^38,52^. Remarkably, the dielectric constant of water decreases from 80.1 (20 °C) down to 19.7 (300 °C) making its solvent properties at ~300 °C roughly comparable with that of acetone at 25 °C^58^ whereas its dissociation constant increases dramatically, leading to more acidic conditions at equivalent pH values. All this facilitates solubilization of organic matter coupled with transformation by ionic condensation, cleavage, and hydrolysis. Under these conditions, water can act as a powerful acid-base catalyst^38^, and ionic reaction pathways could be further facilitated by acidic and basic minerals and dissolved salts. These possible reaction pathways may result in the formation of complex YDOM.

Additionally, above the critical temperature (T_c_ = 374 °C), thermally induced free-radical reactions are likely to become more significant and may even dominate as temperature increases^38^. Water may contribute hydrogen and oxygen for formation of hydrocarbons and oxygenated alteration products^43^. Deep hydrothermal geochemistry near and above supercritical conditions drastically affects hydrogen bonding in aqueous fluids^59^ and might also enable the formation of specific YDOM constituents that are improbable under (near) surface conditions.

Possible geochemical conditions that may be responsible for such a tremendous chemodiversity of YDOM include pressurized hot alkaline-chloride solutions at depth (**RC1**, **OS**), heated H_2_S and H_2_SO_4_ (**EG**), and deep circulating crustal fluids in presence of CaCO_3_ (metamorphozed marine sediments) and H_2_S (**NG**). Thermochemical sulfate reduction processes operating above ~140 °C^43^ further contribute to the observed diversity of CHOS and CHNOS compounds in YDOM.

We conclude (A) that the uniqueness of YDOM was confirmed by compositional comparison with globally collected surface water SPE-DOM samples from rivers, lakes, estuaries, and oceans and by structural and fluorometric comparison with boreal lake SPE-DOM; (B) YDOM complexity cannot be explained by limited biodiversity and expected metabolites, and therefore it must result from its exposure to the inorganic geochemical environment in each spring; (C) YDOM molecular complexity offers a new dimension in defining extreme environments; and (D) YDOM organic chemodiversity can be used to classify springs analogously to the established inorganic geochemical classification currently employed.

## Methods

### Description of the Sampled Hot Springs

The springs reported here are examples of each of these three physiographic types (intra-caldera, caldera-rim, and extra-caldera) and are of different compositional types (alkaline-chloride, mixed-alkaline chloride, acid-chloride-sulfate, and travertine-precipitating), presenting different degrees of connectedness with primary thermal waters, shallow meteoric aquifers, and other crustal fluids. In general, hot springs in the Yellowstone hydrothermal system are considered to show long-term stability of thermal water chemistry^60^, which is also reflected in the reproducible data presented in the HCA in Fig. 1. Figure 5 includes a schematic diagram of the proposed water sources and relative placement of springs within the Yellowstone hydrologic and volcanic system.

The first detailed site, Octopus Spring (**OS**), located within the White Creek Group, Lower Geyser Basin, along with Rabbit Creek 4, Azure Spring, and Ojo Caliente, represents the intra-caldera, high-silica alkaline-chloride hot spring water from the primary hot-water source stored within the rhyolites of the first and third eruption cycles of the Yellowstone caldera. The temperature of the source water for **OS** is ~195 °C, as calculated with the adiabatic quartz geothermometry equation developed by Fournier^51^. This translates to a depth of approximately 130–150 m, following the calculated boiling point curve and by comparison with actual downhole temperatures of the closest drill hole, Y2, located about 1.5 kilometers away^49^. Rhyolites of Central Plateau flows of the first and third eruption cycles host the deep hydrothermal aquifer in this area^49^. The White Creek Group, along with other groups in the Firehole Lake area, comprises several hot springs that are influenced by small amounts of steam condensate, formed when steam, released during subsurface boiling, reaches an impermeable rock layer after which the water condenses. This hot, slightly acidic water reacts with the country rock, leaching trace elements from the minerals. The alkaline-chloride-type water is the same type as that of better-known thermal features, such as Morning Glory Pool, Grand Prismatic Spring, and Old Faithful Geyser^10^. Recent work modeling the regional hydrograph suggests much of the water in the Yellowstone Plateau is recirculated through the hydrothermal system^42^. But some variability in the chemistry of Octopus Spring can be attributed to episodic influx of small amounts of water from an adjacent cold-water marsh, as previously reported^41^; see also supporting online text. **OS** has been the focus of extensive microbiological^14,61^, geochemical^40,62^, and hydrogeological^41,62^ studies.

The second spring, Rabbit Creek 1 (**RC1**) (MRCHSG032, Rabbit Creek Group, Midway Geyser Basin) along with Rabbit Creek 3, discharges water from steam condensate generated by the primary hot-water source mixed with terrestrial input from a small marsh uphill from **RC1** (mixed alkaline-chloride). Organic matter would be exposed to hot mildly acidic conditions, creating background DOM from the milieu of primary sources: terrestrial inputs, microbial inputs, leached soils, and atmospheric inputs. The discharge from the spring is visibly greater than the surface inputs, although measurements of recharge and discharge were not made.

The third spring Elk Geyser (**EG**), along with Cinder Pool, is an example of an acid-chloride-sulfate spring in Norris Geyser Basin (Type III of ^50^), which is located outside the Yellowstone caldera at approximately the intersection of the caldera-rim fracture system and the north-south-trending Norris Mammoth corridor. **EG** was not present in Norris Geyser Basin at the time the White *et al*.^50^ work was conducted. Two major types of hydrothermal water are present at Norris Geyser Basin: alkaline-chloride (Type I of ^50^) and acid-sulfate (Type IV of ^50^). Alkaline-chloride waters are apparently cooled by non-adiabatic processes^42^. Gardner’s *et al*.^42^ comprehensive work does not address the origin of heat and solutes in acid-sulfate or in acid-chloride-sulfate springs. In the classical model, acid-sulfate springs form from the interaction of gases and water vapor, released by subsurface boiling from the underlying magma chamber, with the local meteoric aquifer^10,11^. Carbon dioxide (CO_2_) and hydrogen sulfide (H_2_S) are the dominant gases in such springs. The latter oxidizes to form sulfuric acid, which accounts for the acidity along with carbonic acid from dissolved CO_2_. In the case of acid-sulfate-chloride waters, recirculated alkaline-chloride waters mix with acid-chloride-sulfate waters or are infused with acid gases at depth (hydrothermal with subsurface boiling and hot gas infusion)^63^. Many springs (e.g., Cinder Pool) have detectable quantities of H_2_S along with microbial populations for its oxidation^64^. Other springs, like **EG**, show no evidence of H_2_S, suggesting oxidation takes place in the subsurface. The water in **EG** is isolated from the deep hydrothermal aquifer of the intra-caldera zone by physiographic and hydrological barriers. **EG** is a mixture of extensively acidified, steam-heated shallow aquifer and the more deeply circulating alkaline chloride waters of Norris Geyser Basin^50^. As such, it would have the alkaline-chloride background source of DOM modified through acidic steam extracts.

The fourth spring, Narrow Gauge (**NG**), is in the Mammoth Hot Springs area, north of the Yellowstone Plateau. It is a travertine-precipitating spring. Isotopic evidence suggests the Mammoth system may issue from a separate hydrothermal source from the springs of the Yellowstone Plateau^13^. Source waters for this spring come from deeply circulating crustal fluids that encounter limestone at depth, dissolve CaCO_3_, and become enriched in CO_2_ gas. Such springs are common throughout the northern Rocky Mountains^65^, although none are as extensive as the Mammoth Hot Spring. Upon reaching the surface, CO_2_ exsolves, leading to rapid precipitation of CaCO_3_ in massive travertine terraces. The fluids carry dissolved H_2_S, probably leached from Mesozoic marine sediments. H_2_S is not oxidized in the subsurface and emanates with the carbonate-precipitating fluids. Background organic matter derives from the metamorphosed marine sediments, similar to DOM from alkaline-chloride springs, but recirculation and modification of organic matter derived from this source and modified by contributions from terrestrial and microbial sources appears unlikely due to the relief of the travertine terraces. In fact, most discharge from the Mammoth terraces is fault-controlled and thought to flow from Boiling River^66^, although complete mass balance measurements are not possible. So the DOM associated with Mammoth Hot Springs may be somewhat different from DOM found in acid-chloride-sulfate springs and it appears not to have been exposed to such high temperatures as those achieved by acid-chloride-sulfate springs.

### Sample Collection and Isolation of DOM

Hot spring water samples were collected from selected hot springs in 2010 and 2012. The sampling was undertaken by submerging 2.5 L Pyrex glass bottles into the spring. The 2.5 L glass bottle containing the sample was then allowed to cool below 60 °C before the water was transferred to 20 L glass bottles. This process was repeated until 20 L were collected. The Narrow Gauge spring was sampled using Teflon tubing and spring water was siphoned directly into the 20 L glass containers. 20 L was a minimal requirement to extract sufficient DOM to allow for NMR analyses. All samples were transported to the laboratory located in Yellowstone National Park and then filtered through Whatman GF/F glass fiber filters. A previously described solid-phase extraction procedure^67^ was used to isolate DOM from the water samples using highly efficient (carbon extraction efficiency is about 60%) Agilent Bond Elut PPL solid-phase extraction (SPE) cartridges filled with 1 g of a functionalized styrene-divinylbenzene polymer (PPL) resin. Briefly, after conditioning the Agilent PPL cartridges with 2 cartridge volumes of high purity methanol and rinsing with 1 cartridge volume of acidified ultrapure water (acidified to pH 2), the filtered water samples were acidified (pH 2 with 32% HCl) and gravity-fed to the cartridge. The adsorbed DOM was eluted off the cartridge by using 10 mL high purity methanol and the isolate stored at −20 °C in the freezer prior to FTICR mass spectrometry, NMR spectroscopy and EEM fluorescence analyses.

The following springs were sampled for this study: Octopus Spring, Narrow Gauge, Rabbit Creek (three springs at this location), Elk Spring, Cinder Pool, Ojo Caliente, Azure Spring, and Cinder Pool. To demonstrate the drastic differences of DOM present in a wide variety of common, non-hydrothermal aquatic systems with Yellowstone hot spring DOM, we also collected 1 L freshwater or 10 L seawater samples from 114 sites from diverse aquatic systems: aquatic biomes in New Zealand; the Suwannee River, Georgia, USA (also an IHSS standard reference material); boreal lakes in the Malingsbo area, Arctic lakes in the Abisko area and samples from the Baltic Sea in Sweden; underneath the sea ice in Antarctica, Ross Island; Amazonian rivers and saltwater lagoons in Brazil; the North Pacific and Atlantic Ocean, including Sargasso Sea. DOM from all aquatic systems mentioned above were solid-phase extracted according to the same procedure given above. More details about sampling locations are given in the online supplementary material.

### Characterization of DOM

FT-ICR MS and NMR analyses were undertaken at the Helmholtz Center, Munich, Germany. Mass spectrometric molecular formula assignments and NMR data processing was carried out in a similar fashion as described in previous studies^19,68^. NMR analysis needed to be restricted to a selected set of samples because of the time constraints of analysis. EEM spectra were recorded using a Horiba Aqualog fluorometer at the University of Maryland Center for Environmental Science, Chesapeake Biological Laboratory. Details about each analytical technique are given below.

#### FT-ICR MS

Mass spectra were obtained in negative mode electrospray ionization (ESI) using a Bruker Solarix 12 Tesla FT-ICR mass spectrometer. All methanolic SPE-samples were directly injected into the ionization source at a flow rate of 120 µL min^−1^ and a voltage of 3,600 V. Five hundred transient spectra were averaged at a 4 mega word time domain to yield very accurate and highly resolved *m/z* molecular ions. The high magnetic field and its resulting ultrahigh resolution allowed assigning precise molecular formulas to the majority of observed *m/z* ions. The calculated error between measured and actual mass of assigned formulas was always better than 0.2 ppm. More details about this specific instrument and the associated data analysis have been previously published^19,26^. Double bond equivalency was calculated according to Bae *et al*.^69^.

#### Excitation emission matrix (EEM) fluorescence

One mL of methanolic YDOM and aquatic SPE-DOM samples were dried under ultrapure nitrogen and then re-dissolved in 5 mL ultrapure MilliQ water. EEM spectra were then measured on the aqueous samples using a Horiba Jobin Yvon Aqualog fluorometer at excitation wavelengths ranging from 230–500 nm and emission wavelengths between 200–600 nm. The recorded EEM spectra were then corrected for Raleigh and Raman scattering, inner filtering effect, and normalized to a 1 ppm quinine sulfate standard and expressed in quinine sulfate units (QSU).

#### Nuclear magnetic resonance spectroscopy (NMR)

^1^H NMR spectra of methanolic YDOM extracts were acquired with a Bruker Avance NMR spectrometer at 800.13 MHz (B_0_ = 18.7 T) at 283 K from a few mg of solid obtained by evaporation of original methanol solution, dissolved in approx. 130 µL CD_3_OD (Merck, 99.95% ^2^H) solution with a 5 mm z-gradient ^1^H/^13^C/^15^N/^31^P QCI cryogenic probe (90° excitation pulses: ^13^C~^1^H~10 µs) in sealed 2.5 mm Bruker MATCH tubes. 1D ^1^H NMR spectra were recorded with a spin-echo sequence (10 µs delay) to allow for high-Q probe ringdown, and classical presaturation to attenuate residual water present “*noesypr1d”* (5 s acquisition time, 5 s relaxation delay, 1 ms mixing time; 1 Hz exponential line broadening). A phase sensitive, gradient enhanced echo-antiecho TOCSY NMR spectrum with solvent suppression (*dipsi2etgpsi19*) was acquired with an acquisition time of 1 s, a mixing time of 70 ms, and a relaxation delay of 1 s (spectral width of 9615.4 Hz, computed to a 16384 × 2048 matrix. The one bond coupling constant ^1^J(CH) used in 2D ^1^H,^13^C DEPT-HSQC spectra (*hsqcedetgpsisp2*.*2*) was set to 145 Hz; other conditions: ^13^C 90 degree decoupling pulse, GARP (70 µs); 50 kHz WURST 180 degree ^13^C inversion pulse (Wideband, Uniform, Rate, and Smooth Truncation; 1.2 ms); F2 (^1^H): spectral width of 9572.2 Hz (11.96 ppm); 1.25 s relaxation delay; F1 (^13^C): SW = 40252 Hz (200 ppm); 36224.9 Hz (180 ppm, for **OS** and **RC1**). HSQC-derived NMR spectra were computed to a 4096 × 512 matrix. Gradient (1 ms length, 450 µs recovery) and sensitivity enhanced sequences were used for all 2D NMR spectra. Similarity of ^1^H NMR spectra (Supplementary Fig. S4) was computed from 0.001 ppm section integrals in the range δ_H_ = 0.5–9.5 ppm, with exclusion of methanol and residual water (Bruker AMIX software, version 3.9.4.) with Hierarchical Cluster Explorer (HCE); similarity versus distance metrics used Pearson correlation coefficients. Other NMR acquisition conditions are given in Supplementary Table. S5.

#### Statistical Analyses

Stiff diagrams were used to visualize the inorganic composition of selected springs. Inorganic parameters used for Hierarchical Cluster Analysis (HCA) are summarized in Supplementary Table S1. All HCA were undertaken on auto-scaled data^70^ using Pearson correlations and average linkage. Simple absence presence analyses were used to isolate unique molecular formulas for the YDOM and SPE-DOM sample sets.

## Electronic supplementary material

Supplementary Material
