# Supplementary material for: Yellowstone Hot Springs are Organic Chemodiversity Hot Spots
Source: Sci Rep. 2018 Sep 20;8:14155. doi: 10.1038/s41598-018-32593-x (PMC6147864; doi:10.1038/s41598-018-32593-x)
Supplement: Supplementary file 1 — Supplementary Material [file 41598_2018_32593_MOESM1_ESM.pdf]

Supplementary Online Material

Yellowstone Hot Springs are Organic Chemodiversity Hot Spots

Authors: Michael Gonsior[1*], Norbert Hertkorn[2], Nancy Hinman[3], Sabine E.-M. Dvorski[2], Mourad Harir[2], William J. Cooper[4], and Philippe Schmitt-Kopplin[2,5*]

Affiliations:

[1]University of Maryland Center for Environmental Science, Chesapeake Biological Laboratory, Solomons, Maryland, USA.

[2]Helmholtz Zentrum Muenchen, German Research Center for Environmental Health, Neuherberg, Germany.

[3]University of Montana, Department of Geosciences, USA.

[4]University of California Irvine, Department of Civil and Environmental Engineering, USA.

[5] Technische Universität München, Institute for Analytical Food Chemistry, Freising-Weihenstephan, Germany.

*Correspondence to:
Michael Gonsior, email: gonsior@umces.edu; phone: 410-326-7245.
Philippe Schmitt-Kopplin, email Schmitt-kopplin@helmholtz-muenchen.de; phone: 089-3187-3246

Structural diversity of YDOM as seen by NMR spectroscopy. High-field NMR spectroscopy provides the capability for quantitative and non-destructive *de novo* determination of chemical environments from polydisperse and molecularly heterogeneous environmental samples, such as DOM. Quantitative relationships between number of spins and area (1D NMR) and volume (2D NMR) of NMR resonances operate in the absence of differential NMR relaxation, which is more pronounced in 2D NMR than in 1D NMR experiments [1]. These quantitative relationships in NMR spectroscopy augment complementary structure-selective analytical methods, like mass spectrometry (which detects gas phase ions and is subject to ionization selectivity in the case of complex mixtures [2]), and fluorescence spectroscopy (which selectively detects fluorescent chemical environments of sp^2-hybridized carbon [3]).

NMR spectroscopy is particularly informative in the description of aliphatic chemical environments, which are based on sp^3-hybridized carbon. These are inactive in fluorescence spectroscopy, and the difference in size of aliphatic groups will cause rather inconspicuous mass shifts in FT-ICR mass spectra; more extensive aliphatic systems, i.e. longer aliphatic carbon chains, will result in higher mass molecules, with somewhat larger H/C (closer to 2.0) and lower O/C elemental ratios. This characteristic is however insufficient to allow reliable conclusions about chemical structures. NMR spectroscopy enables distinction of the size of aliphatic units but also allows for in-depth assessment of its intrinsic chemical environments, like open chain and cyclic arrangements of carbon [4]. Open-chain, branched aliphatic compounds eventually terminate in methyl groups, which show discernible NMR resonances at $\delta_H < 1$ ppm, whereas cyclic aliphatic environments will resonate between $\delta_H \sim 1$-2 ppm, depending of ring size, ring fusion, and ring conformation [5]. The effects of aliphatic branching on δ_H and δ_C differ considerably; marginal aliphatic effects on δ_H beyond directly bonded carbon contrast with aliphatic increments on δ_C up to three bonds away for any C-C bond (δ_C increments for carbon substitution Cα: +9.1 ppm; Cβ: +9.4 ppm; Cγ: -2.5 ppm; Cδ: 0.3 ppm). These relationships allow reconstruction of key aliphatic substructures in molecularly heterogeneous and polydisperse DOM from homo- and heteronuclear 2D NMR spectra [6].

The positioning of the $\underline{\mathbf{H_3C}}$-C cross-peaks in heteronuclear single quantum coherence (HSQC) NMR spectra (Fig. S6) results from main operating effects: (i) alkyl substitution in the α-position (H_3C-C_α-; three different options of C-substitution) increases δ_C; (ii) alkyl substitution

in the β-position (H_3C-C-C_β-; ten different options of C-substitution) decreases δ_C. Alicyclic rings show greater short-range connectedness than do open-chain aliphatic compounds; this leads to a more effective transmission of remote effects (which are commonly associated with downfield chemical shift) on δ_H and δ_C.

All ^{1}H NMR spectra of YDOM exhibit more distinct patterning than those of boreal lakes (Fig. S4). This does not reflect less diversity of chemical environments in YDOM but rather different arrangements of chemical bonds. **RC1** and **NG** are largely characterized by open-chain aliphatic compounds and contain smaller proportions of oxygen-containing functional groups than does **OS**; **EG** features abundant carboxylic groups and lesser proportions of oxygenated aliphatic groups (OCH_n-units) in comparison with other YDOM.

However, ^{1}H, ^{1}H total correlation spectroscopy (TOCSY) NMR spectra of **RC1** and **NG** share several major cross-peaks among YDOM samples (Fig. S5), suggesting the presence of similar basic branched aliphatic motifs. Discernible “detached” methyl resonances with $\delta_H < 1$ ppm, which represent extended branched aliphatic systems, decrease in relative amplitude according to **RC1** > **NG** > **OS** > **EG** (Fig. S4, Fig. S5). This implies that the average size of aliphatic environments in **NG** is larger than that in **RC1**.

Both **RC1** and **NG** contain mainly open-chain aliphatic compounds whereas **OS** and especially **EG** are primarily composed of alicyclic units, like carboxyl-rich alicyclic acids (CRAM); this fundamental distinction cannot be retrieved from any other analysis (Fig. S4, Fig. S5a, Fig. S6a). Partitioning of one-dimensional ^{1}H NMR spectra by HCA and PCA at 0.001 ppm bin resolution, which accounts for intrinsic dissimilarities of atomic-resolution substituents, differentiates open-chain aliphatic substituents of **RC1** from those of **NG** (Fig. S4a, Fig. S4b) whereas partitioning of the same ^{1}H NMR spectra at 0.1 ppm bin resolution, which indicates the distribution of bulk aliphatic branching motifs, is less distinctive.

In **EG**, the ^{1}H, ^{1}H TOCSY cross-peak amplitude is significantly influenced in relation to the signal to noise ratio of 1D ^{1}H NMR spectra, for both aliphatic (Fig. S5a) and aromatic (Fig. S5b) chemical environments. This attenuation results from differential transverse NMR relaxation and is likely caused by extensive metal coordination of appropriate **EG** molecules. CRAM-related CHα-units (HOOC-**CHα**-CHβ-; δ_H: 2.0 – 2.7 ppm), which are likely candidates for metal coordination of their carboxyl groups, are particularly affected. These presumably

organo-metal interactions appear abundant in the 1D ^{1}H NMR spectra of **EG** but are severely alleviated in TOCSY cross-peak amplitude. The comparison of respective NMR resonances in **OS** and **EG** indicates that analogous metal coordination will be largely absent in **OS** (Fig. S5).

Aromatic ($C_{ar}H$) units (δ_H: 9 – 6.5 ppm) in YDOM fell into two groups and were also particularly distinct from boreal lake SPE-DOM (Fig. S5b, Fig, S6b). **EG** showed near Gaussian distribution of aromatic NMR resonances, suggesting an even abundance of electron-withdrawing (COX: COOH, COOR, -C=CR; including a substantial proportion of polycarboxylated aromatic rings (δ_H > 7.5 ppm), neutral (C_{alkyl}, H), and electron-donating functional groups (-OR, -OH) (Fig. S4, Fig. S5b). A ramp-like increase of aromatic hydrogen with decreasing chemical shift δ_H, rarely observed in common freshwater SPE-DOM, indicated abundant polyphenolic compounds and/or aromatic ethers in the order **OS >> RC1** > **NG**, suggesting variable contributions from terrestrial organic matter and plant phenolic input (Fig. S4, Fig. S5b, Fig. 6b). Effects of distal substitution on aromatic protons as obtained from TOCSY NMR spectra also revealed fundamental differences in aromatic chemical environments of YDOM (Fig. S5b). The proportions of $C_{ar}O$-units (δ_H < 7 ppm for hydrogen in ortho- and para- positions) co-varied with those of aliphatic hydrogen next to carbon-oxygen bonds **H**CO-groups (δ_H ~3.5 – 3.9 ppm) in **NG**, **RC1,** and **OS** (Fig. S4), corroborating the presence of plant polyphenols. However, YDOM from **OS** showed not only much higher proportions of these groups but also a remarkably distinct chemodiversity, with absence of the sharp NMR resonance of aliphatic methyl esters (Fig. S4; δ_H: ~ 3.64 ppm), substantial abundance of aromatic methyl ethers (Fig. S7), and presence of various oxygenated aromatic compounds (Fig. 5b, Fig. 6b), with resemblance to common lignin derivatives (Fig. 6b) [7,8]. While oxygenated $C_{ar}O$ units are clearly present in **EG**, the respective cross-peaks at δ_H < 7 ppm (Fig. 5b, Fig. 6b) may as well arise from aromatic ethers (cf. Fig. S7), further indicating diverse functional groups in YDOM.

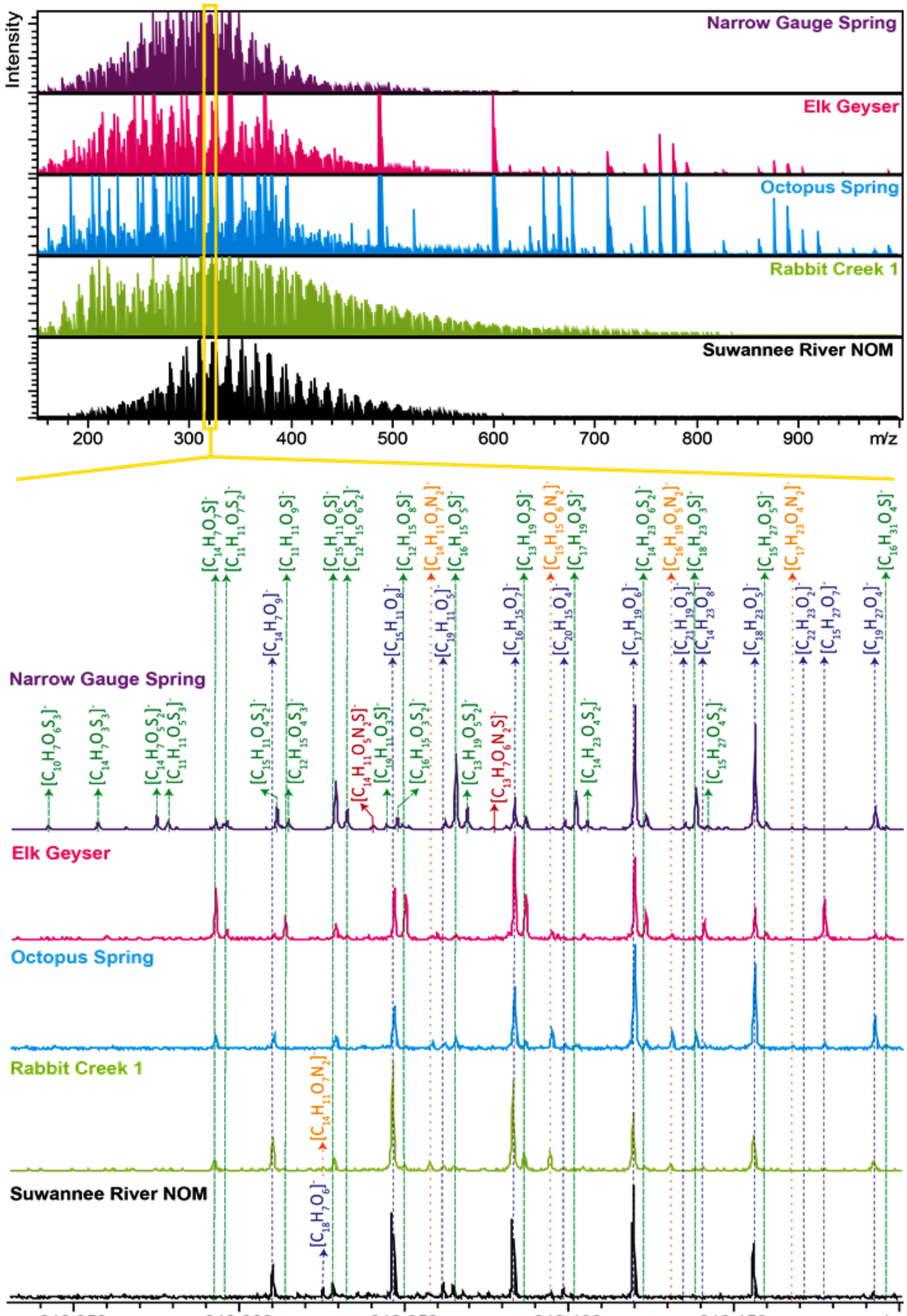

Fig. S1. Negative electrospray FTICR mass spectra of four YDOM (Narrow Gauge Spring **NG** (purple), Elk Geyser **EG** (pink), Octopus Spring (light blue) and Rabbit Creek 1 **RC1** (light green) in comparison with a Suwannee River reference material NOM (2R101N; black). Zoomed area of nominal mass 319 (m/z = 318.93 – 319.20), with color-coded assignment according to CHO, CHNO, CHOS and CHNOS molecular series.

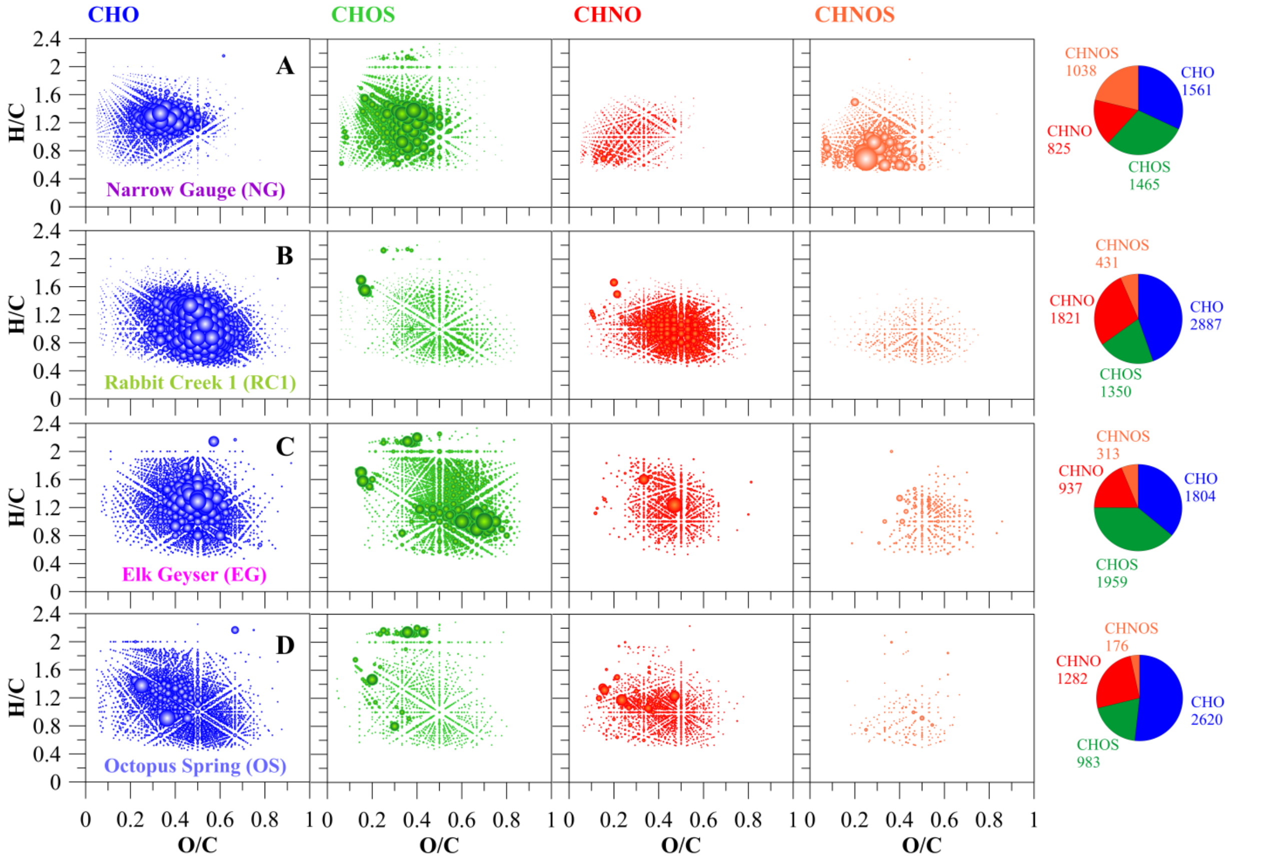

Fig. S2. Van Krevelen diagrams for four YDOM; (A) Narrow Gauge Spring **NG**, (B) Elk Geyser **EG**, (C) Octopus Spring **OS** and (D) Rabbit Creek 1 **RC1**; color code: CHO (blue), CHOS (green), CHNO (red), CHNOS (orange) molecular series. Note: bubble size reflects relative abundance of m/z ions.

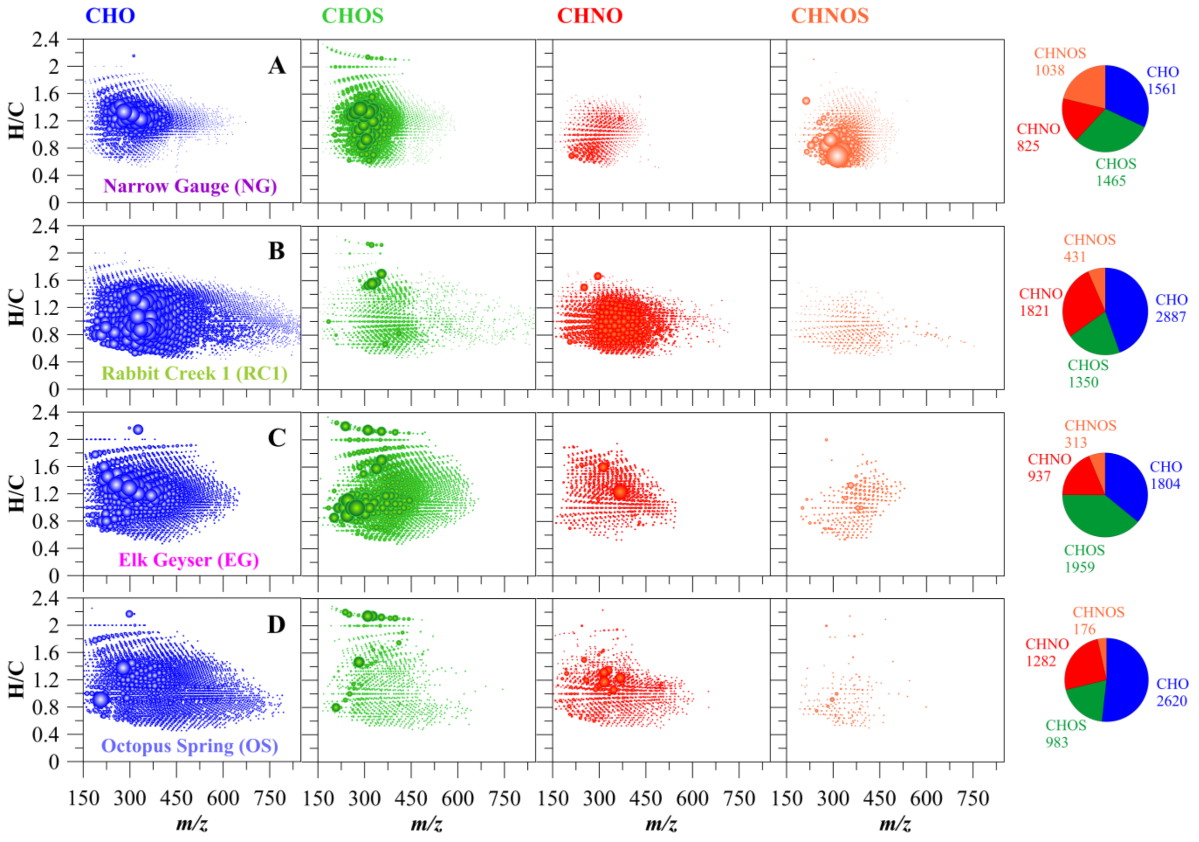

Fig. S3. Mass-resolved H/C ratios for four YDOM; (A) Narrow Gauge Spring **NG**, (B) Elk Geyser **EG**, (C) Octopus Spring **OS** and (D) Rabbit Creek 1 **RC1**. Color code according of CHO (blue), CHNO (orange), CHOS (green) and CHNOS (red) molecular series; bubble area reflects relative abundance of m/z ions. Formula distributions (pie diagrams) repeated from Fig. S2.

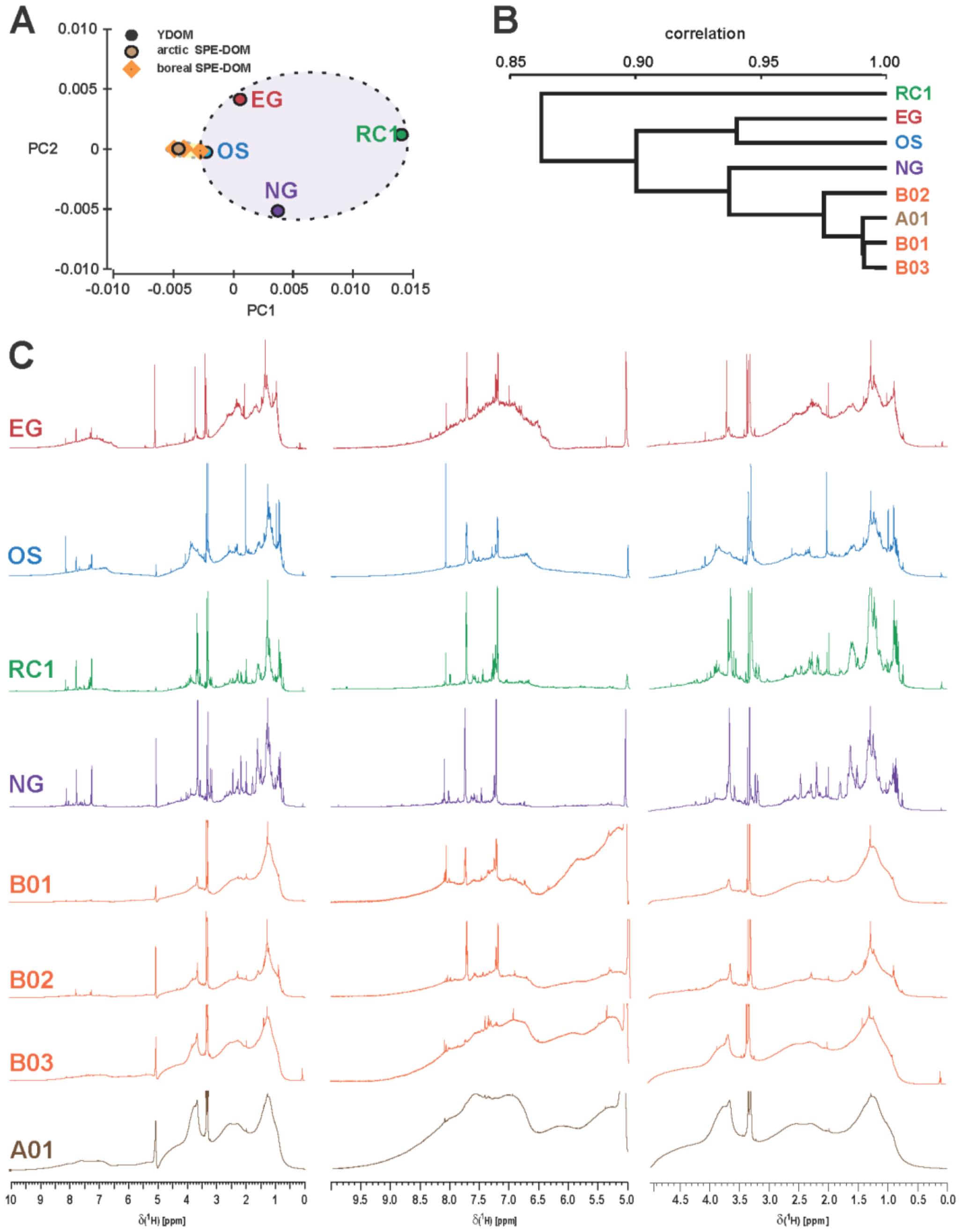

Fig. S4. (A) Principal component analysis (PCA) and (B) hierarchical cluster analysis (HCA) of ^{1}H NMR spectral data (800 MHz, CD_3OD) of specific hot springs and a set of 8 SPE-DOM samples collected from Swedish lakes.. (C) ^{1}H NMR spectra of the four YDOM samples and Swedish lakes (cf. main text). Note: The central and right ^{1}H NMR spectra are zoomed in to the different areas of chemical shifts.

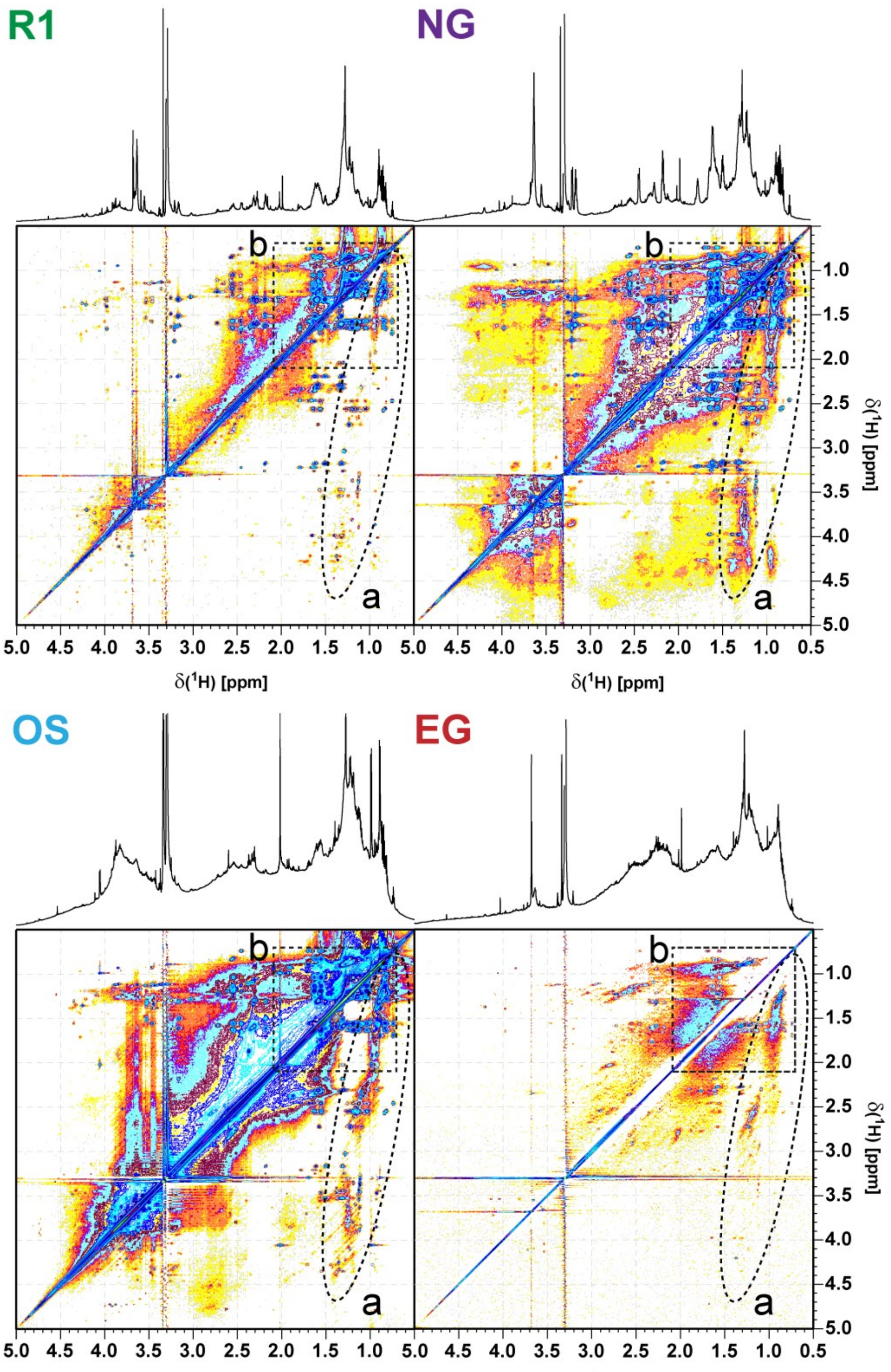

Fig. S5a. ^{1}H, ^{1}H TOCSY NMR spectra of four YDOM (800 MHz, CD_3OD), indicating correlations in aliphatic (-Y-$\underline{H}C_{sp}{}^3$-C_n-$C_{sp}{}^3\underline{H}$-Z-; n = 0-2; Y, Z = C, O, N, S) spin systems (cf. main text); circled area a: ($\underline{\mathbf{H_3C}}$-$C_n$-$C_{sp}{}^3\underline{\mathbf{H}}$-Z-; n = 0-2; Z = C, O, N, S); area b: -C-$\underline{\mathbf{H}}C_{sp}{}^3$-$C_n$-$C_{sp}{}^3\underline{\mathbf{H}}$-C-; n = 0-2.

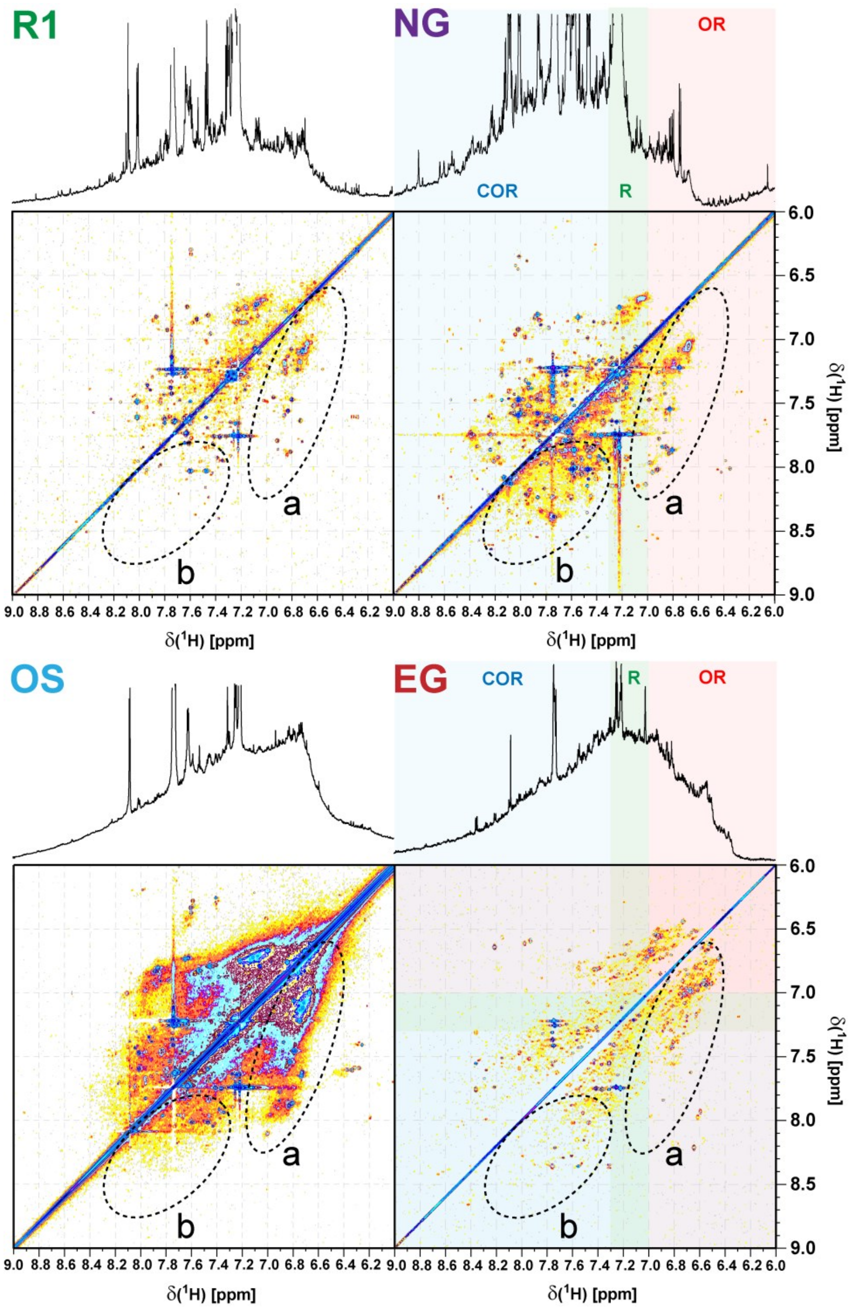

Fig. S5b. ^{1}H, ^{1}H TOCSY NMR spectra of four YDOM (800 MHz, CD_3OD), indicating correlations in aromatic ($\underline{H}C_{sp2}$-C_n-$C_{sp}{}^{2}\underline{H}$; n = 0-2) spin systems (cf. main text). circled area a: $\underline{\mathbf{H}}C_{sp2}$-$C_n$-$C_{sp2}\underline{\mathbf{H}}$-O-; n = 0-2; circled area b: $\underline{\mathbf{H}}C_{sp2}$-$C_n$-$C_{sp2}\underline{\mathbf{H}}$-(C=O)-; n = 0-2. Note: Shaded background colors provided in selected spectra refer to ortho- and para-substitutions in aromatic rings. Red shade: electron-donating substituents (δ_H < 7 ppm), green shade: electro-neutral substituents (δ_H: 7.0 – 7.43 ppm), blue shade: electron-withdrawing substituents (δ_H > 7.3 ppm).

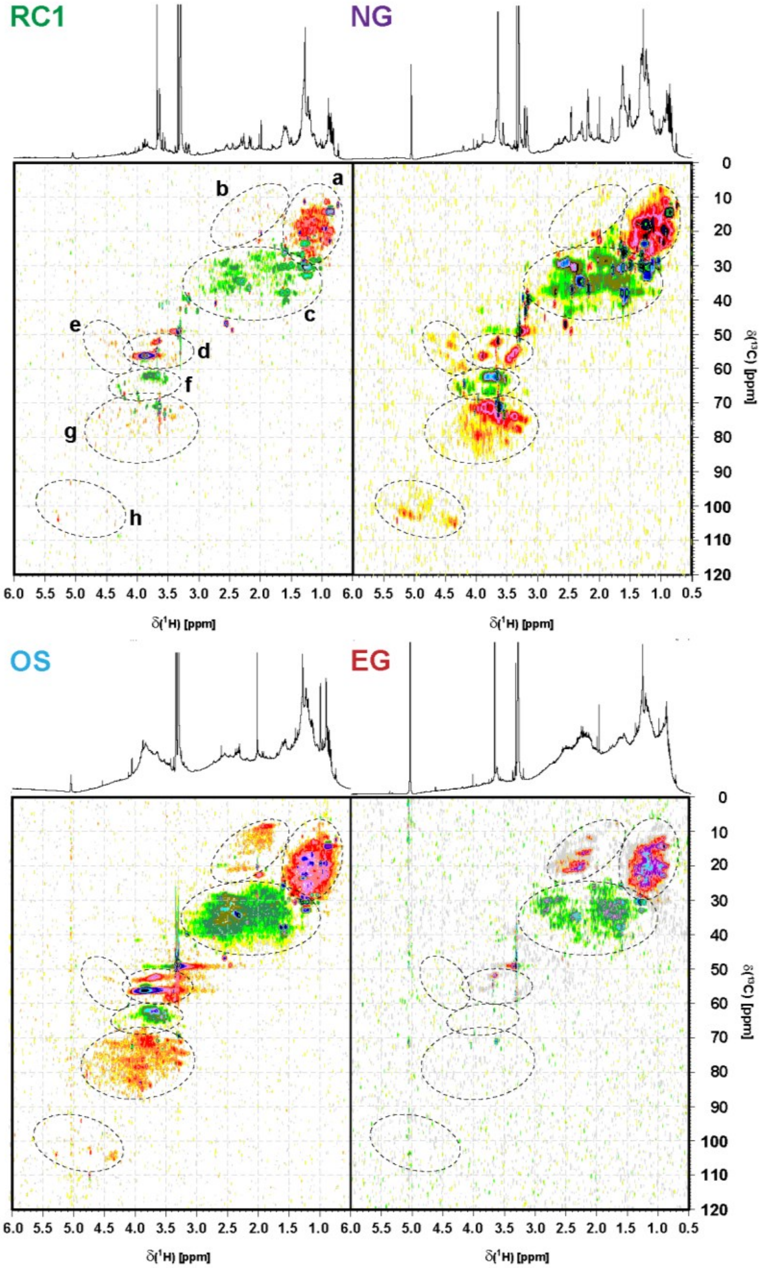

Fig. S6a. Carbon multiplicity-edited ^{1}H, ^{13}C HSQC NMR spectra (800 MHz, CD_3OD; CH and CH_3: red; CH_2: green) of four YDOM: aliphatic [$^1J(C_{sp}{}^3H)$] correlations; relevant substructures as indicated: area a: C-$\underline{CH_3}$; area b: =C-$\underline{CH_3}$ and –S-$\underline{CH_3}$; area c: HOOC-C_n-$\underline{CH_2}$-, $\delta_H > 2.2$ ppm: n = 0; $\delta_H < 2.2$ ppm: n ≥ 1); area d: OCH_3 (Fig. S7); area e: -C=O-NH-$\underline{C_\alpha H}$- in peptides; area f: O$\underline{CH_2}$ (δ_H : 62/3.5-3.8 ppm); area g: C_3-$\underline{CH}$; area h: O_2-$\underline{CH}$-C.

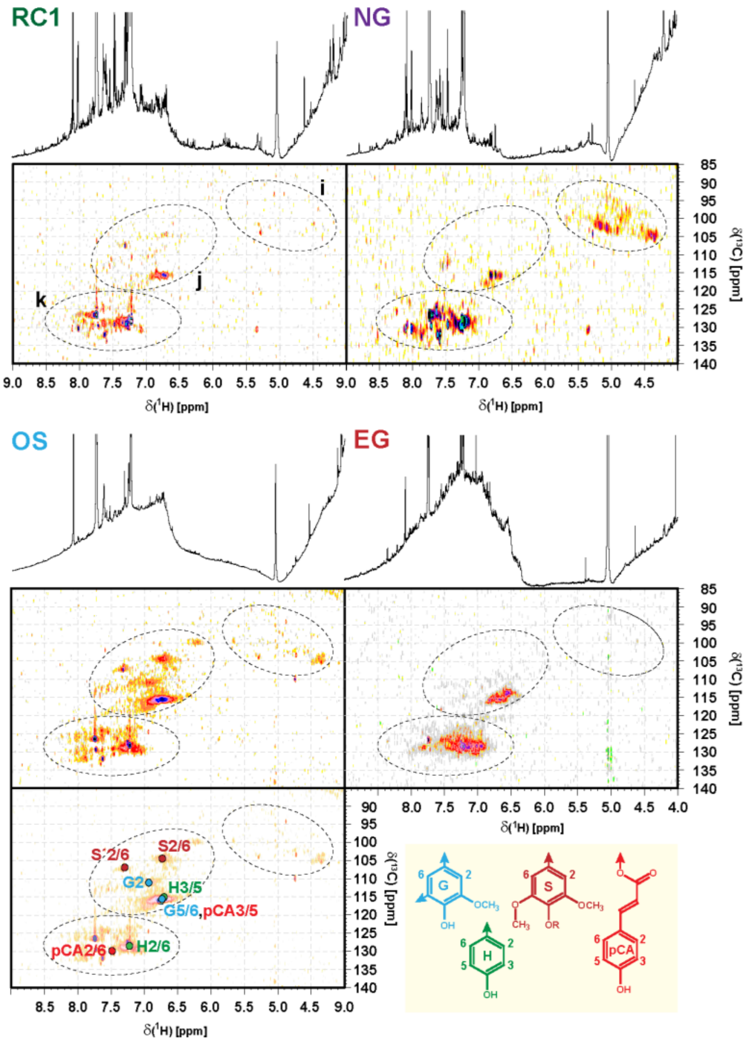

Fig. S6b. Carbon multiplicity-edited ^{1}H, ^{13}C HSQC NMR spectra (800 MHz, CD_3OD; CH: red) of four YDOM: aromatic [($^{1}J_{sp2}H$)] correlations; relevant substructures as indicated: area i: O_2-CH-C (anomerics in carbohydrates); area j: electron-donating aromatics: oxygen in *ortho* and *para* positions; common aromatics, at δ_H > 7.3 ppm, electron withdrawing COX (X: OH, OR, R). For Octopus Springs **OS**, tentative cross peak assignment according to common lignin substructures (G: guaiacyl units; S: syringyl units, S´: syringyl unit with OCH_3 at C4-position; H: p-hydroxyphenyl units; pCA: p-coumarate units). Rabbit Creek **RC1** also shows weak analogous lignin-derived cross peaks.

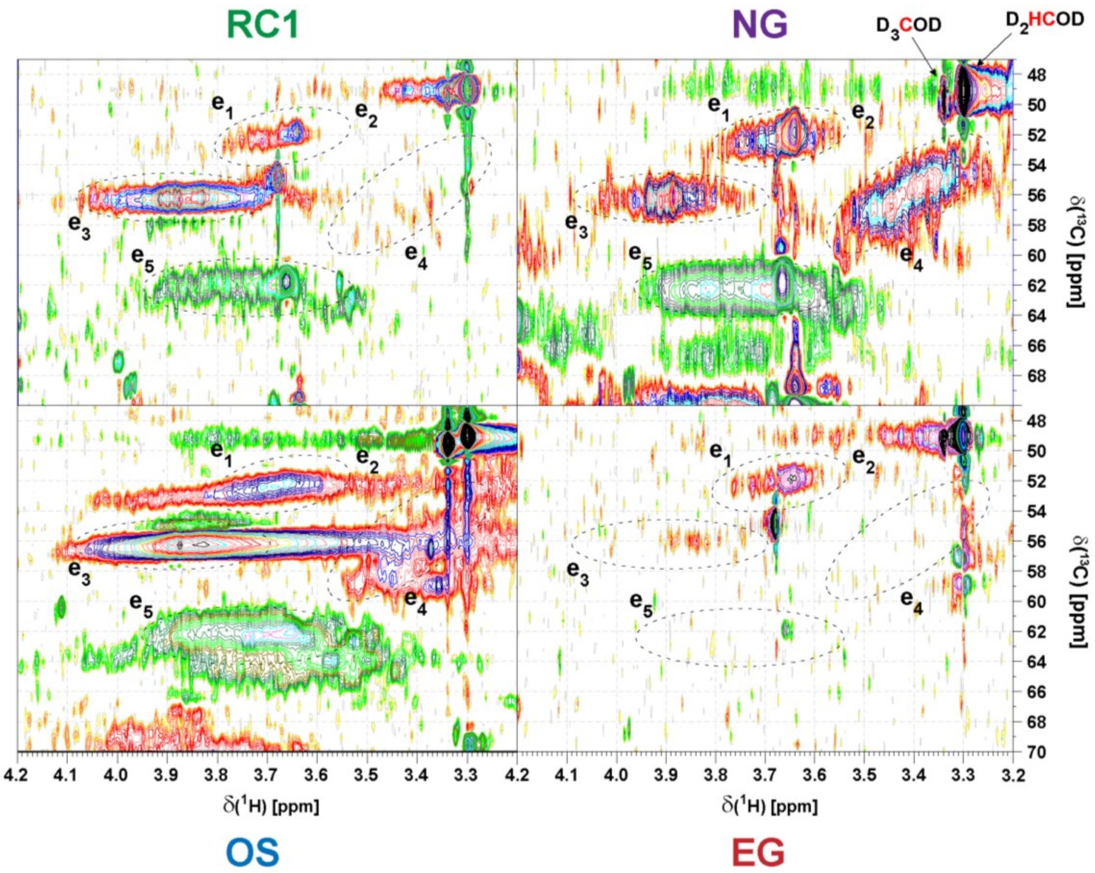

Fig. S7. Carbon multiplicity-edited ^{1}H, ^{13}C HSQC NMR spectra of four YDOM (800 MHz, CD_3OD; CH and CH_3: red; CH_2: green); section of $O\underline{CH_3}$ cross peaks and anomeric carbon ($O\underline{CH_2}$), with distinct ranges of $\delta_{H/C}$ of aromatic (e_1) and aliphatic (e_2) methyl esters; aromatic (e_3) and aliphatic methyl ethers (e_4) as well as (e_5) oxomethylene ($O\underline{CH_2}$), likely associated with carbohydrates.

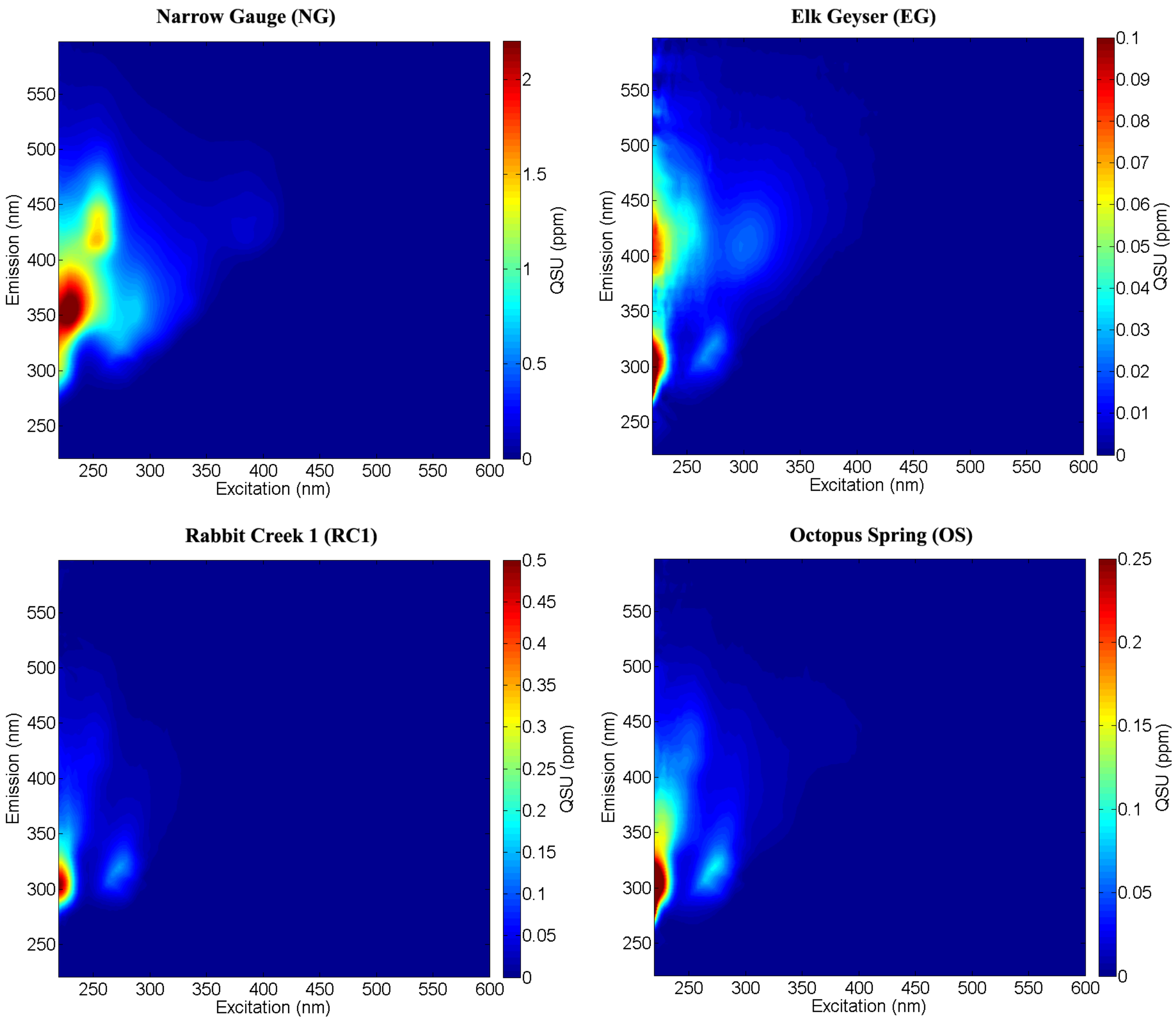

Fig. S8. EEM fluorescence spectra of Narrow Gauge (**NG**), Rabbit Creek 1 (**RC1**), Elk Geyser (**EG**) and Octopus Spring (**OS**). Note: 1 ml methanol extracts were dried and re-dissolved in 5 mL ultrapure Milli-Q water.

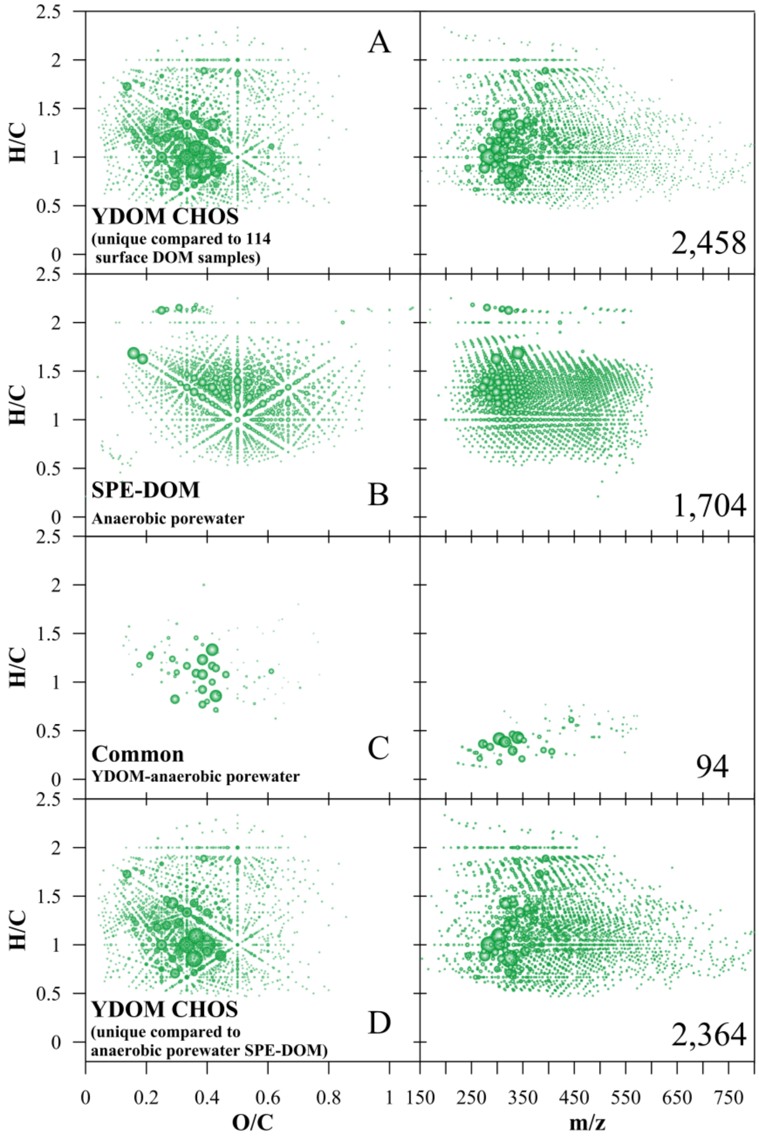

Fig. S9. Van Krevelen diagrams (left) and mass-edited H/C ratios (right) of consolidated CHOS compounds in the set of four YDOM that was unique when compared to an analogous consolidated set of 114 aerobic aquatic SPE-DOM samples and that of an anaerobic sediment pore water from Chesapeake Bay, USA. (A) Set of unique CHOS molecular compositions found in four YDOM when contrasted with the consolidated CHOS molecular compositions found in 114 aerobic aquatic SPE-DOM samples. (B) Anaerobic sediment pore water SPE-DOM sample collected at 10 cm depth of a sediment core from the Chesapeake Bay, USA (cf. main text). (C) Common CHOS molecular compositions (ne = 94) found in a set of four consolidated YDOM contrasted with anaerobic sediment pore water from Chesapeake Bay, USA. (D) Set of unique YDOM CHOS formulas after the comparison with the anaerobic porewater SPE-DOM.

Table S1. Inorganic characteristics of Elk Geyser (**EG**), Narrow Gauge (**NG**), Octopus Spring (**OS**) and Rabbit Creek 1 (**RC1**).

item	unit	detection limit	NG	EG	RC1	OS
**T**[1]	°C		66	76	79	89
**pH**[2]			7.3	3.5	7.1	7.9
**DO**[3]	mg/L	0.0	0.0	5.2	3.6	3.2
**H_2S**[3]	mg/L	0.011	0.20	bdl	bdl	bdl
**F**	mg/L	0.05	3	7	19	19
**Cl**	mg/L	0.6	160	646	257	245
**SO_4^{2-}**	mg/L	0.5	524	80	19	16
**Na**	mg/L	0.05	131	413	312	309
**Si**	mg/L	0.1	23	152	101	115
**Al**	µg/L	50	70	1395	400	380
**Fe**	µg/L	50	bdl	112	bdl	bdl
**Mn**	µg/L	5	23	5	bdl	bdl
**Mo**	µg/L	10	bdl	195	20	20
**HCO_3^-**	mmol/L	0.05	21.6	bdl	7.5	9.9
**Ca**	mg/L	0.05	350.6	7.57	0.8	0.5
**Mg**	mg/L	0.05	72.00	bdl	0.16	0.05

1. ± 1˚C. 2. ± 0.3 pH units. 3. ± 5 %. Error for ICP and IC analyses ± 10 %. bdl = below detection limit.

Table S2. Counts of peaks as computed from negative electrospray FT-ICR mass spectra for single charged ions with nitrogen rule checked.

members of molecular series	NG	EG	OS	RC1
**CHO formula counts**	1561 (20.8%)	1804 (34.4%)	2622 (49.7%)	2887 (43.3%)
**CHOS formula counts**	3207 (42.6%)	1968 (37.5%)	1007 (19.1%)	1360 (20.4%)
**CHNO formula counts**	847 (11.3%)	1097 (20.9%)	1336 (25.3%)	1837 (27.6%)
**CHNOS formula counts**	1906 (25.3%)	374 (7.1%)	311 (5.9%)	583 (8.7%)
**total**	**7521**	**5243**	**5276**	**6667**
**average *m/z***	330.3	339.7	337.5	375.7
**Percent mass**				
**average H [%]**	6.1	6.5	6.9	5.8
**average C [%]**	59.8	57.3	62.3	59.0
**average O [%]**	24.2	29.8	23.8	30.9
**average N [%]**	0.8	1.3	3.7	1.2
**average S [%]**	9.2	5.1	3.3	3.1
**percent atoms**				
**average H [%]**	47.1	48.5	49.5	45.2
**average C [%]**	38.5	35.7	37.2	38.3
**average O [%]**	11.7	13.9	10.7	15.1
**average N [%]**	0.4	0.7	1.9	0.7
**average S [%]**	2.2	1.2	0.7	0.8
**H/C_w**	1.22	1.36	1.36	1.18
**O/C_w**	0.30	0.39	0.29	0.39
**C/N_w**	87.2	51.4	19.6	57.4
**C/S_w**	17.3	30.0	50.3	50.7
**$(DBE)_w$**	7.5	6.4	7.4	8.7
**$(DBE/O)_w$**	1.7	1.2	2.0	1.4
**$(\#C)_w$**	16.5	16.3	17.6	18.5
**$(DBE/C)_w$**	0.5	0.4	0.4	0.5

Note: The suffix $_w$ means average intensity weighted values. DBE: double bond equivalence; DBE/O: DBE divided by number of oxygen atoms; #C: average number of carbon in assigned formulas; DBE/C: DBE divided by number of carbon atoms.

Table S3. Descriptive FTICR MS analysis of consolidated DOM (114 aquatic samples) and hot spring DOM (10 hot spring samples (at least two from each spring from different years).

item	consolidated SPE-DOM from 114 different aquatic environments					consolidated SPE-DOM from 10 hot springs (YDOM)			
	**CHO**	**CHOS**	**CHNO**	**CHNOS**		**CHO**	**CHOS**	**CHNO**	**CHNOS**
**n**	5603	4744	6981	4869		4159	5430	3511	2895
**average *m/z***	418.9	409.9	443.2	451.4		353.8	342.1	343.9	341.5
**O/C_w**	0.48	0.45	0.47	0.44		0.40	0.34	0.36	0.34
**H/C_w**	1.24	1.43	1.19	1.51		1.20	1.38	1.16	1.06
**DBE_w**	8.7	6.2	10.0	6.5		8.2	6.0	8.9	8.6
**DBE/O_w**	0.9	0.8	1.1	0.9		1.2	1.2	2.0	1.9
**$\#C_w$**	20.0	18.2	20.4	18.4		18.1	16.0	17.1	14.6
**DBE/C_w**	0.4	0.3	0.5	0.4		0.5	0.4	0.5	0.6

Note: The suffix $_w$ means average intensity weighted values. DBE: double bond equivalence; DBE/O: DBE divided by number of oxygen atoms; #C: average number of carbon in assigned formulas; DBE/C: DBE divided by number of carbon atoms.

Table S4. Reported thermophile communities in Yellowstone hot springs.

Spring	Genus species	Order	Reference
Elk Geyser (**EG**)	No data available	No data available	
Octopus Spring (**OS**)	Thermocrinus sp., Hydrogenobacter thermophiles, Aquifex pyrophilus and aeolicus, Thermoproteales pyrobaculum	Aquificales, Crenarchaeota and Sulfulobales	[9-12]
Narrow Gauge (**NG**)	Exiguobacterium	Bacillales	[13]
Rabbit Creek 1-4 (**RC1-4**)	No data available	No data available	
Cinder Pool (**CP**)	Hydrogenobaculum	Aquificales	[14]
Azure Spring (**AS**)		Aquificales, Thermatogales	unpublished
Ojo Caliente (**OC**)	Thermatogales, Thermocrinis, Thermoproteales and Candidatus Acetothermia	Aquificales, Sulfulobales, Desulfurococcales and Cadidatus Acetothermia	[15]

Table S5. Acquisition parameters of NMR spectra, shown according to figures. NS: number of scans (for 2D NMR: F2); AQ: acquisition time [ms]; D1: relaxation delay [ms]; NE: number of F1 increments in 2D NMR spectra; WDW1, WDW2: apodization functions in F1/ F2 (EM/GM: line broadening factor [Hz]; QS: shifted square sine bell; SI: sine bell); PR1, PR2: coefficients used for windowing functions WDW1, WDW2, EM/GM are given in [Hz], SI/QS derived functions indicate shift by π/n.

**Spectrum**	**Figure**	**NS**	**AQ [ms]**	**D1 [ms]**	**NE**	**WDW1**	**WDW2**	**PR1**	**PR2**
**NG**	S5	1024	8100	11900	-	-	EM	-	1
**EG**	S5	1600	8300	6700	-	-	EM	-	1
**RC1**	S5	1280	5000	15000	-	-	EM	-	1
**OS**	S5	1280	8300	15000	-	-	EM	-	1
**NG**	S6	40	1000	1000	2048	QS	EM	2.5	2.5
**EG**	S6	20	1000	1000	1600	QS	EM	2.5	2.5
**RC1**	S6	32	1000	1000	2048	QS	EM	2.5	2.5
**OS**	S6	40	1000	1000	2048	QS	EM	2.5	2.5
**NG**	S7	1024	250	1250	202	QS	EM	2.5	2.5
**EG**	S7	1280	250	1250	286	QS	EM	2.5	2.5
**RC1**	S7	1024	250	1250	292	QS	EM	2.5	2.5
**OS**	S7	640	250	1250	360	QS	EM	2.5	2.5

Table S6. ^{1}H NMR section integrals of key substructures (800 MHz, CD_3OD) from YDOM.

δ(^{1}H) [ppm]	**key substructures**	**NG**	**EG**	**RC1**	**OS**
10-6.5 ppm	C_{ar}**H**	5.8	12.4	10.5	12.2
6.5 - 5.15 ppm	=C**H**, O_2C**H**	0.2	0.4	0.6	1.3
4.9 - 3.1 ppm	OC**H**	21.1	10.5	20.9	21.2
3.1 - 1.9 ppm	OCC**H**	21.6	31.6	20.5	25.1
1.9 - 0 ppm	CCC**H**	51.3	45.2	47.4	40.2

Table S7. Detailed ^{1}H NMR section integrals (800 MHz, CD_3OD) from YDOM (for annotation of chemical shift regions, see Dvorski et al., 2016).

δ(^{1}H) [ppm]	**NG**	**EG**	**RC1**	**OS**
10 - 7.3 ppm	4.0	6.6	5.9	6.6
7.3 - 7.0 ppm	1.4	2.8	2.8	2.4
7.0 - 6.5 ppm	0.4	3.0	1.8	3.2
6.5 - 6.0 ppm	0.1	0.3	0.5	1.2
5.3 - 5.15 ppm	0.2	0.1	0.1	0.1
4.9 - 3.1 ppm	21.1	10.5	20.9	21.2
3.1 - 2.1 ppm	17.5	25.7	16.7	20.1
2.1 - 1.9 ppm	4.1	5.9	3.8	5.0
1.9 - 1.35 ppm	20.3	18.9	15.9	15.2
1.35 - 1.25 ppm	9.2	5.7	10.6	5.4
1.25 - 0 ppm	21.9	20.5	20.9	19.7

References

1 Hertkorn, N. in *eMagRes* (John Wiley & Sons, Ltd, 2007).

2 Hertkorn, N. *et al.* Natural Organic Matter and the Event Horizon of Mass Spectrometry. *Analytical Chemistry* **80**, 8908-8919, doi:10.1021/ac800464g (2008).

3 Lakowicz, J. R. & Masters, B. R. Principles of Fluorescence Spectroscopy, Third edition. *Journal of Biomedical Optics* **13** (2008).

4 Hertkorn, N. *et al.* Characterization of a major refractory component of marine dissolved organic matter. *Geochim. Cosmochim. Acta* **70**, 2990-3010 (2006).

5 Günther, H. *NMR umfassend: „NMR-Spektroskopie. Grundlagen, Konzepte und Anwendungen der Protonen- und Kohlenstoff-13- Kernresonanzspektroskopie in der Chemie”*□. . 3 edn, Vol. 41 (Thieme Verlag, 1992).

6 Hertkorn, N., Harir, M., Koch, B. P., Michalke, B. & Schmitt-Kopplin, P. High-field NMR spectroscopy and FTICR mass spectrometry: powerful discovery tools for the molecular level characterization of marine dissolved organic matter. *Biogeosciences* **10**, 1583-1624, doi:10.5194/bg-10-1583-2013 (2013).

7 Kim, H. & Ralph, J. Solution-state 2D NMR of ball-milled plant cell wall gels in DMSO-d6/pyridine-d5. *Organic & Biomolecular Chemistry* **8**, 576-591, doi:10.1039/b916070a (2010).

8 del Río, J. C. *et al.* Structural Characterization of Wheat Straw Lignin as Revealed by Analytical Pyrolysis, 2D-NMR, and Reductive Cleavage Methods. *Journal of Agricultural and Food Chemistry* **60**, 5922-5935, doi:10.1021/jf301002n (2012).

9 Huber, R. *et al.* Thermocrinis ruber gen. nov., sp. nov., a Pink-Filament-Forming Hyperthermophilic Bacterium Isolated from Yellowstone National Park. *Applied and Environmental Microbiology* **64**, 3576-3583 (1998).

10 Jahnke, L. L. *et al.* Signature Lipids and Stable Carbon Isotope Analyses of Octopus Spring Hyperthermophilic Communities Compared with Those ofAquificales Representatives. *Applied and Environmental Microbiology* **67**, 5179-5189, doi:10.1128/aem.67.11.5179-5189.2001 (2001).

11 De La Torre, J. R., Walker, C. B., Ingalls, A. E., Könneke, M. & Stahl, D. A. Cultivation of a thermophilic ammonia oxidizing archaeon synthesizing crenarchaeol. *Environmental Microbiology* **10**, 810-818, doi:10.1111/j.1462-2920.2007.01506.x (2008).

12 Jay, Z. J. *et al.* The distribution, diversity and function of predominant Thermoproteales in high-temperature environments of Yellowstone National Park. *Environmental Microbiology*, n/a-n/a, doi:10.1111/1462-2920.13366 (2016).

13 Vishnivetskaya, T. A., Kathariou, S. & Tiedje, J. M. The Exiguobacterium genus: biodiversity and biogeography. *Extremophiles* **13**, 541-555, doi:10.1007/s00792-009-0243-5 (2009).

14 Spear, J. R., Walker, J. J., McCollom, T. M. & Pace, N. R. Hydrogen and bioenergetics in the Yellowstone geothermal ecosystem. *Proc. Natl. Acad. Sci. U. S. A.* **102**, 2555-2560, doi:10.1073/pnas.0409574102 (2005).

15 Meyer-Dombard, D. A. R. *et al.* Hydrothermal ecotones and streamer biofilm communities in the Lower Geyser Basin, Yellowstone National Park. *Environmental Microbiology* **13**, 2216-2231, doi:10.1111/j.1462-2920.2011.02476.x (2011).

269
